# Application of a Dynamic Exposure Population Toxicokinetic Model for Perfluorooctane Sulfonic Acid (PFOS) and Extension to Perfluorodecanoic Acid (PFDA) at a North American Beef Cattle Farm with a History of Biosolids Land Application

**DOI:** 10.3390/toxics13070541

**Published:** 2025-06-27

**Authors:** Barbara A. Astmann, Antti T. Mikkonen, Thomas L. Simones, Meghan Flanagan, Duncan Pfaehler, Ivan Lenov, Andrew E. Smith

**Affiliations:** 1 Maine Center for Disease Control and Prevention, Augusta, ME 04333, USA; barbara.a.astmann@maine.gov (B.A.A.); thomas.simones@maine.gov (T.L.S.); 2 Environment Protection Authority (EPA) Victoria, Centre for Applied Sciences, Macleod, VIC 3085, Australia; antti.mikkonen@epa.vic.gov.au; 3 Department of Agriculture, Conservation and Forestry, Augusta, ME 04333, USA; mlf45@cornell.edu (M.F.); duncan.pfaehler@maine.gov (D.P.); 4 U.S. Department of Agriculture Food Safety and Inspection Service, Saint Louis, MO 63120, USA; ivan.lenov@usda.gov

**Keywords:** perfluorooctane sulfonic acid (PFOS), perfluorodecanoic acid (PFDA), biosolids, cattle, dynamic exposure, toxicokinetics, body burden, food safety, risk management

## Abstract

Historical application of wastewater treatment sludge (biosolids) has introduced per- and polyfluoroalkyl substances (PFAS) into agricultural systems and led to contamination of crops and livestock. Previous work validated a dynamic exposure and population toxicokinetic (DE_PopTK) modeling approach for estimating perfluorooctane sulfonic acid (PFOS) and perfluorohexane sulfonic acid (PFHxS) concentrations in cattle tissues at sites primarily dominated by water contamination. This work expands the efforts to validate the DE_PopTK model at a self-contained beef farm in Maine with PFAS exposures from feed grown on site where soil is contaminated from historical biosolids applications. The model is also extended to estimate perfluorodecanoic acid (PFDA) exposure and tissue levels. Farm-specific data were obtained to consider farm management practices, spatial variation of PFAS in soil, animal growth, and seasonal and annual variability in estimating daily exposures based on water, feed, and soil intake. A dynamic exposure pattern was observed as cattle accumulated PFAS while consuming feed grown on contaminated land and eliminated it while grazing on non-contaminated pastures. Model-estimated PFOS and PFDA levels in serum and muscle were in good agreement with biomonitoring data collected at the farm over a four-year period to reflect periods of accumulation and depuration, with the percentage error ranging from 16% to 73% when comparing modeled and measured data. Our findings demonstrated that understanding farm exposures and collecting site-specific data were integral to model performance. The model was applied to simulate management strategies and complement economic analyses to demonstrate that, with modifications to management practices, it is feasible for the farm to achieve lower PFOS and PFDA levels in beef and maintain economic viability despite elevated PFAS soil levels.

## 1. Introduction

Per- and polyfluoroalkyl substances (PFAS) are a class of chemicals with widespread use in industrial processes, food packaging, and various consumer products. PFAS have been detected in numerous waste streams, including wastewater treatment sludges, also referred to as biosolids [1,2,3,4,5,6]. The land application of biosolids in agriculture is a common practice in many countries [7,8]. The long-term practice of biosolids land application as a soil amendment has introduced PFAS into the soils of farmlands across the United States (U.S.) [4,9,10]. Based on analysis of archived U.S. biosolids from the 2001 U.S. Environmental Protection Agency (EPA) National Sewage Sludge Survey, the estimated mean load of thirteen PFAS applied to U.S. agricultural land was 1375 to 2070 kg (kg) per year [5]. In the state of Maine, systematic statewide testing of farm fields with a history of biosolids land application has found soil levels of perfluorooctane sulfonic acid (PFOS) ranging from <1 to over 1000 nanograms per gram (ng/g) [11].

PFAS-contaminated soils have been linked to the contamination of livestock tissues through the grazing and feeding of forage crops [12,13,14,15,16,17]. Tests of commercial food supplies as part of total diet surveys have reported generally low levels of PFAS contamination [18,19,20]. Exposures can be higher in localized settings where there is known PFAS contamination and consumption of locally sourced animal products is more likely [21,22,23,24]. In some cases, the discovery of PFAS contamination on agricultural land and in livestock has led to extreme measures, including the depopulation of contaminated herds and loss of farming livelihoods [25,26].

Assessing local PFAS exposure in agricultural settings and whether it can be mitigated at contaminated farms poses a challenge for risk assessment. Standard risk assessment modeling approaches for PFAS typically assume constant exposures and steady-state kinetics [27]. However, a farm may have considerable spatial variability in soil PFAS levels among fields and variability in PFAS plant uptake from soil by forage crops among fields on the same farm [14]. Additionally, livestock PFAS exposure may be influenced by substantial seasonal and behavioral patterns associated with farming practices [16]. This potential for substantial changes in exposure to legacy PFAS such as PFOS, combined with an apparent half-life on the order of two to three months for PFOS in milk and beef tissues, can result in significant temporal changes in tissue levels over the course of a year [12,16,28,29]. Mikkonen et al. [30] developed an integrated dynamic exposure and population toxicokinetic (DE_PopTK) model for beef cattle that estimates serum and tissue concentrations of PFOS and perfluorohexane sulfonic acid (PFHxS) over time. Exposures were estimated from daily intakes of water, pasture, and soil and modeled as a function of growth over the lifespan of an animal. Modeling allowed for temporal variability (water and forage intake) and variable PFAS concentrations across fields used for grazing. This model was validated against monitoring data from Australian farms where exposures were largely dominated by contaminated water, with one site having appreciable variation in exposure as cattle were rotated on and off a field with contaminated water and soil. The model was also modified to simulate the exposure of a dairy herd in Sweden using data on PFAS levels in feed and water to reflect low-level exposure presumably from background contamination [30]. For these exposure scenarios, the DE_PopTK model was used to evaluate farm management practices for reducing PFAS exposure, such as cattle rotation or targeted supplementation with clean feed and water. The model outputs for exposure management scenarios demonstrated opportunities for marked reductions in consumer exposures from beef products [30].

In this study, we report the application of a modified version of the Mikkonen et al. [30] DE_PopTK model (the full R code can be found in the supplementary information) to a beef cattle farm in the northeastern U.S., where exposure is dominated by a contaminated feed pathway presumably resulting from the historical application of biosolids. The cattle at this farm experienced pronounced seasonal differences in PFAS exposure resulting from grazing on pastures with low or non-detectable PFAS soil levels from late spring to early fall, then consuming contaminated stored feed harvested from fields with elevated PFAS soil levels for the remainder of the year. A major aim of this study was to validate the model for exposures dominated by contaminated soil and plant exposure pathways in a setting with appreciable spatial variation in soil PFAS levels. We also extended the DE_PopTK model to perfluorodecanoic acid (PFDA) exposures. We validated the model against measured PFOS and PFDA serum and muscle tissue levels in beef cattle that were born and raised on the farm, with nearly all feed sourced onsite. In addition, we applied the model to test options for farm management practices for reducing PFAS exposure and maintaining economic viability for this farm.

## 2. Materials and Methods

### 2.1. Description of Farm Environment and Practices

The study site is a self-contained small-scale beef farm located in central Maine with over 100 acres used for pasture and livestock forage and approximately 20–25 head of Angus cattle that are born and raised on the farm. The farm is a grass-based operation that feeds grass-based forages grown onsite, including pasture grasses and stored feed defined herein as dry hay and silage (grass-based forage preserved via fermentation). The farm also provides some supplemental grain in the final months before cattle go to slaughter. The farm’s well serves as the primary drinking water source for cattle via troughs available within housing and pastures.

**Figure 1:**
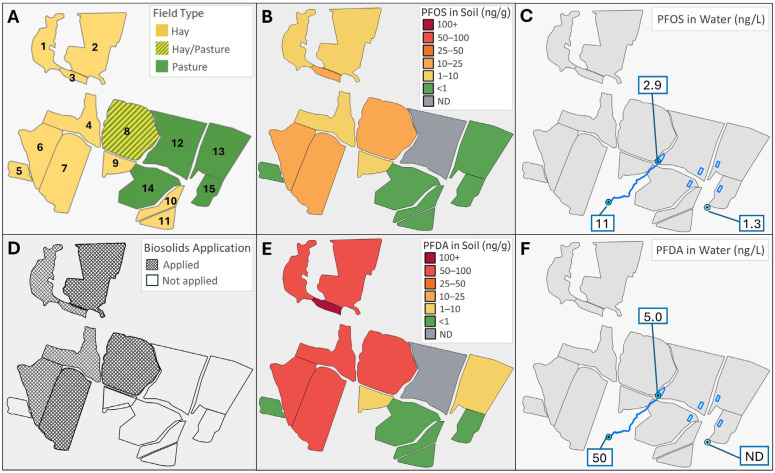
Site overview map with (**A**) numbered farm fields by use, (**B**) fieldwide composite soil PFOS concentrations, (**C**) PFOS concentrations measured in untreated well water and two surface water bodies accessible from pastures, (**D**) biosolids application history by field, (**E**) fieldwide composite soil PFDA concentrations, and (**F**) PFDA concentrations measured in untreated well water and two surface water bodies accessible from pastures.

From 1983 to 2019, roughly 11,500 cubic meters of biosolids, sourced from four different municipal wastewater treatment facilities, were recorded to have been applied to the farm fields. Some fields used to grow hay (and historically corn silage) received several thousand cubic meters of biosolids, while other fields received only hundreds of cubic meters. There was no licensed application of biosolids on the farm’s pasture fields. A dynamic exposure pattern was expected, as cattle typically cycled through an approximately 6-month period of PFAS accumulation when consuming stored feed and a 6-month depuration period while grazing (hay fields and pastures shown in Figure 1A).

Exposure to PFAS begins in utero via maternal transfer, and following calving, exposures are predominantly related to diet. Calves and breeding stock are typically on pasture between June and October, where calves are nursing while transitioning to consuming pasture forage. Calves are then weaned and fed stored feed for the next 6 months, generally from November to May, until returning to pasture mid-May. Cattle are either sold live at auction at the end of the following summer or are held through another winter of consuming stored feed and slaughtered the following spring. 

### 2.2. Site PFAS Characterization

All environmental sampling followed the Maine Department of Environmental Protection (Maine DEP) standard operating procedure for PFAS sampling [31]. Farm field soils were sampled using three methods for obtaining field-wide composite samples that evolved over the course of the study to improve exposure estimates of fieldwide PFAS soil levels. For pasture fields, samples were obtained using 10-part composite sampling in a dispersed pattern; for hay fields, samples were obtained using either 15- or 30-part composite sampling using a gridded sampling method. For all soil composite sampling methods, soil core samples were collected to a depth of 15–20 centimeters (cm) using a 1.9 cm diameter stainless steel push probe (AMS, American Falls, ID, USA). Soil cores were hand homogenized in stainless steel bowls with visible rocks, plant material, and biota removed. Subsamples were taken from each bowl, put into sample containers, immediately placed in coolers with ice, and frozen until shipment for analysis. More detail on the soil sampling methods and a map displaying the type of composite sampling by field are provided in the Supplementary Materials (Figure S1).

In July 2022 and June 2023, co-located soil and plant samples were collected from three hay fields to obtain site-specific PFAS soil-to-plant transfer factors (these data were published as Farm C in Simones et al. [14]). Additionally, between July and September 2023, cut hay samples were collected from five fields just after being cut and raked into rows by the farmer in preparation for baling. Fifteen evenly spaced grab samples were collected from each sample area (sample areas shown in Figure S1). The grab samples were composited, placed in a polyethylene bag, kept in a cooler with ice immediately following collection, and frozen until shipment for analysis.

The farm well, a pond, and a stream were sampled in accordance with Maine DEP’s standard operating procedures for water sample collection from water supply wells and surface water and sediment sampling [32,33].

### 2.3. Biomonitoring

All sampling of cattle serum and/or muscle tissue was performed either on farms or at a licensed slaughterhouse. Animals were cared for and managed by farmers, and all blood and tissue sampling was undertaken with the permission of the farm owner. All animal sampling was conducted for the primary purpose of understanding the chemical contamination of food animals. All procedures on live animals were completed by or under the direct supervision of a licensed veterinarian with the primary goal of maintaining animal welfare. All sampling plans for deceased animals were carried out directly following humane euthanasia by a veterinarian or humane slaughter by trained slaughterhouse personnel.

The biomonitoring data used for model evaluation were exclusive to the study site, with muscle and/or blood samples collected from four groups of cattle over a four-year period. Biomonitoring data collected at an additional three sites were utilized for model parameterization. A summary of all the biomonitoring work is provided in Table 1, and additional details are provided in the Supplementary Materials.

Blood was collected from either the jugular or coccygeal vein using vacutainer tubes (Becton, Dickinson and Company, Franklin Lakes, NJ, USA) with anticoagulant for plasma and without anticoagulant for serum. Serum samples were allowed to clot at room temperature, while plasma samples were refrigerated or frozen until further processing. Both sample types were centrifuged at 1500 revolutions per minute for 12 min to separate the clot or cells from the supernatant. The plasma or serum was collected, frozen, and shipped for chemical analysis. Plasma results were converted to serum equivalent results using a ratio determined from paired serum and plasma samples (provided in Supplementary Materials Table S15). Both live and postmortem muscle tissue sampling was conducted. Live muscle biopsy samples were collected in 2.5 × 10 cm strips, weighing at least 5 g per sample, from the external and/or internal abdominal oblique muscles from the flank area of each animal. At slaughter, 5–10 g samples were collected from the flank regions of the animals. Muscle tissue samples were placed into polyethylene bags, placed in a cooler with ice, and frozen until shipment for analysis.

### 2.4. PFAS Analyses

All samples were shipped on ice to a commercial or governmental laboratory for PFAS analyses. The methods for PFAS analysis in soil, water, and plant tissue are described in a previous publication [14]. Briefly, soil, surface water, and well water samples were sent to Pace (Westborough, MA, USA), where 28 PFAS compounds were measured via a lab-developed isotope dilution targeted analysis method. PFAS concentrations in soil were reported in ng/g on a dry weight basis. Cut hay samples were sent to the U.S. Food and Drug Administration (FDA) Center for Food Safety and Applied Nutrition (College Park, MD, USA), where 30 PFAS compounds were measured using the FDA method C-010.02 [34,35]. For animal tissues, Battelle (Norwell, MA, USA) used their DoD QSM 5.3 Table B-15 method or the U.S. EPA 1633 method for targeted analysis of 28 and 40 PFAS compounds, respectively. The U.S. Department of Agriculture (USDA) Food Safety and Inspection Service (St. Louis, MO, USA) measured PFAS in serum, plasma, and muscle samples using their Screening, Determination, and Confirmation of PFAS method CLG-PFAS 2.03 [36,37]. Additional USDA method details are provided in the Supplementary Materials. Quality control measures were followed, as specified by each laboratory/method. In addition, interlaboratory comparisons were performed to ensure comparability of results and are described in the Supplementary Materials.

### 2.5. Exposure Model

The dynamic exposure (DE) model described in Mikkonen et al. [30] was used to model exposure on a per day basis. The model used temporal (dry matter content of feed, temperature and precipitation), spatial (contaminant concentrations), farm practice (field rotation, feed supplementation, age at weaning, use of first-cut versus second-cut hay), and physiological (body weight, forage and water consumption rates linked to body weight) variables to estimate daily intakes and doses. Weather data were downloaded from U.S. National Oceanic and Atmospheric Administration National Centers for Environmental Information, and mean daily precipitation and temperature data were calculated for weather stations in the Central Maine region from 10 May 2019, through 31 October 2024 [38]. Available farm records and management practices informed exposure model inputs for each group of cattle sampled (described in Table 1), with each modeled exposure scenario described in Table 2.

In the absence of farm records detailing the order of feeding hay bales by field of origin, stored feed exposure to PFAS was modeled as a weighted-average feed concentration for each year’s crop. First- and second-cut hay exposures were modeled separately, as plant uptake of PFAS has been shown to be greater in second-cut grasses [14,39]. For first-cut hay, field-specific PFAS feed concentrations were calculated by multiplying each individual field’s measured soil concentration by a site-specific plant transfer factor to estimate PFAS in the feed harvested from each field. The average feed PFAS concentration for an annual crop was then calculated as the sum of the estimated individual field feed concentrations weighted by the fraction of bales from each field relative to the total number of bales harvested that year. To calculate the average PFAS concentration for second-cut hay, the process described above was modified to weight by the fraction of second-cut bales from each field relative to the total number of second-cut bales harvested, and increase the site-specific transfer factor 2.7-fold for PFOS and 2.1-fold for PFDA based on the observed higher plant transfer factor in second-cut grasses reported by Simones et al. [14] and described in Table S3. Farm records informed the timing of feeding second-cut hay relative to first-cut hay (described in further detail in the Supplementary Materials).

For stored feed exposures in the 2024 model scenario, PFAS feed concentrations were estimated from the measured plant tissue levels obtained from the sampling of cut hay from five fields in the 2023 growing season, representing 78% of the total crop that year. Feed from the remaining unsampled fields was modeled using the plant transfer factor method as described above. For the average 2024 stored feed concentration, both measured and estimated PFAS feed concentrations by field were weighted by the fraction of bales from each field relative to the total number of bales harvested for the 2023 growing season.

PFAS exposure from grazing was modeled using the soil concentrations measured in each pasture, the site-specific plant transfer factors to estimate PFAS in pasture forage, the pasture rotation schedule, and the estimated time spent on each pasture. Except for Field 8 (Figure 1A), the time spent on each pasture was not provided by the farmer, therefore the number of days spent on all other pastures was estimated based on relative acreage. Field 8 is a hay field that is also used as pasture for a 6-week period in the fall when cattle are less than a year old. Because grazing typically occurred on Field 8 following at least one cutting, pasture forage concentrations were assumed to be representative of a second cutting and estimated using the adjusted second-cut plant transfer factors. Incidental soil ingestion was considered while cattle were grazing at a soil intake rate of 4% dry matter intake based on the U.S. EPA’s soil ingestion rate utilized in a risk assessment model for beef cattle raised on contaminated soil [40].

PFAS exposure from water was modeled based on concentrations measured in well water and the farmer’s observation of cattle preferentially using troughs filled with well water over surface water in pastures. In February 2022, an activated carbon filtration system was installed for the farm well. Prior to the filter installation, exposure to water was estimated based on low-level measured concentrations (Figure 1). After filter installation, it was assumed that there was no ongoing exposure to PFOS and PFDA from well water, as concentrations were below detection or reporting limits. Additional details related to estimating exposure from stored hay, grazing, and drinking water are provided in the Supplementary Materials.

A modification was made to the exposure equations previously published by Mikkonen, et al. [30] to include milk intake for calves, as described in Equations (1) and (2). Calf milk intake was estimated by calculating cow milk yield using a published mature beef cow milk production model:

(1) $${MY}_{t}=\frac{\left[A\times {\left(t+14\right)}^{b}\times e^{\left(-c\times (t+14)\right)}\right]\times YP}{10}$$

where *MY_t_* is milk yield at time *t* (kg/day), *t* is days after the calf is born, *YP* is the peak milk production rate adjusted for breed (8.16 kg/day), and *A*, *b*, and *c* are model parameters calculated based on day of peak milk yield (*A* = 1.69, *b* = 0.57, and *c* = 0.009 when peak milk yield occurs on day 48 post calving) [41,42,43,44]. Calf milk intake is converted from total milk volume to dry matter intake assuming a milk dry matter content of 13% [45]. Nursing calves consume an increasing amount of forage as they grow, dependent on milk consumption and forage quality [44]. Dry matter intake from forage was modified from Mikkonen, et al. [30] to consider nursing calf forage intake relative to milk intake using Equation (2) [44].

(2) $${DMI}_{F}=\frac{\left(0.0783\times BW-4.87\times {DMI}_{M}\right)}{{DE}_{F}}$$

where *DMI_F_* is dry matter intake of forage (kg/day), *BW* is calf body weight (kg), *DMI_M_* is the dry matter intake of milk (kg/day), and *DE_F_* is forage digestible energy (value of 2.895 megacalories per kg selected as the mean of *DE_F_* values for grasses provided by National Research Council [46]). Equation (2) was used to calculate DMI_F_ until a specified time of weaning, after which *DMI_F_* is calculated using the method described in Mikkonen, et al. [30]. Daily milk concentrations were generated from estimating daily PFAS doses from forage, soil, and water as the input for an initial run of the DE_PopTK model to simulate breeding stock animals, with milk concentrations estimated using serum/milk partition coefficients (Pmilk) (Table 3) to then be used as the inputs for milk exposure for calves in each exposure scenario.

### 2.6. Toxicokinetic Model

The population toxicokinetic (PopTK) model was adopted from Mikkonen, et al. [30]. Briefly, the PopTK model utilizes a first-order one-compartment model, with serum concentrations reflecting the concentration of PFOS or PFDA in this compartment. The model predicts the concentrations of PFOS or PFDA in serum based on modeled daily intake of PFAS from exposure, volume of distribution (Vd), and serum half-life (DT50). The one compartment model is extended to predict muscle or milk concentrations via the use of empirically derived partition coefficients for PFOS and PFDA that relate the concentration in serum to that in tissue under the assumption of a constant ratio between serum and tissue concentrations. The overall model structure was unaltered, with only minor modifications to expand the dataset previously used to compute a meta-mean PFOS PM value to include paired serum and muscle data collected at four Maine farms (Figure S2).

To extend the PopTK model to PFDA, modifications were made to the parameter input file to include PFDA toxicokinetic parameters. The PFDA DT50 is based on data reported by Drew et al. [29], while PM is the meta-mean of paired cattle muscle–serum data from four Maine farms (Figure S2). In the absence of PFDA-specific data and considering the similarity between PFOS and PFDA in half-life and muscle partitioning, the PFOS parameter values for Vd, Pmilk, and maternal transfer utilized in Mikkonen et al. [30] were applied for PFDA. A sensitivity analysis was performed to evaluate model performance when assuming a PFDA Vd that was 2-fold lower or higher than the Vd for PFOS while also assuming a constant coefficient of variance in stochastic simulations. The simulation analysis indicated that the Vd for PFDA in beef cattle is likely to be within a factor of two of the Vd for PFOS (Figure S3). The toxicokinetic parameter inputs selected for PFOS and PFDA are provided in Table 3.

### 2.7. Model Evaluation and Statistical Analyses

Cattle lifespans were modeled using site-specific exposure information described above, with separate runs for each year in which biomonitoring was conducted. One hundred stochastic simulations were performed to account for individual heterogeneity in toxicokinetic parameters and variation in feed and water intake. Each simulation used a different random draw from normal distributions for the toxicokinetic parameters; means and standard deviations are presented in Table 3. Additionally, predicted feed and water intakes were allowed to vary by 30%, as described by Mikkonen et al. [30]. For each model run, the mean and standard deviation of the 100 simulations were calculated and compared to the mean and standard deviation of the measured muscle and/or serum data from the relevant biomonitoring year. Model results are presented graphically to illustrate variation among individual simulations and overlaid with the observed biomonitoring data and reference lines for published action levels for PFOS in beef muscle. The upper reference line at 3.4 ng/g refers to the beef action level derived by the state of Maine in 2020 [47]. The lower line at 0.3 ng/g refers to the European Union (EU) maximum limit published in 2022 [48]. Action levels do not yet exist for PFDA in beef muscle.

Computations of weighted-average PFAS concentrations in feed, means and standard deviations of biomonitoring data, and percentage errors comparing model estimates to biomonitoring data were performed in Microsoft Excel. Meta-analyses of muscle/serum partition coefficients, DE_PopTK model runs, and the computation of means and standard deviations across 100 simulations per model run were implemented in RStudio (version 2025.05.1), building upon the sample code provided in Mikkonen et al. [30]. An updated sample DE_PopTK R code is included in the Supplementary Materials.

### 2.8. Farm Management Analyses

The study farm’s business model has historically been to send 24-month-old cattle to slaughter in late spring and sell meat directly to consumers. An economic analysis was performed to assess two farm management options to attain lower muscle PFAS levels prior to market: (1) purchasing uncontaminated feed to be fed during the second winter prior to slaughter and (2) selling animals at approximately 18 months of age at live auction following 6 months grazing on pastures with low or non-detectable PFAS levels. Live cattle weights were estimated using growth equations described by Mikkonen et al. [30]. The total meat weight per animal at a slaughter age of 24 months was estimated to be 578 pounds, assuming 63% hanging weight and subtracting 17.5% of the hanging weight to account for the weight of bones [49,50]. National average retail price data per pound of 100% ground beef were obtained for the years 2020–2024 to determine the gross price per animal [51]. The cost of purchasing hay and grain was based on prices reported by the USDA National Agricultural Statistics Service (NASS) and farm records for purchased grain [52]. These costs were subtracted from the gross price per animal to determine the net profit per animal. Data provided by the USDA NASS for monthly average auction prices for feeder cattle from 2020 through 2024 were used to calculate the average price per pound of the live animal at auction [53]. Each yearly average was multiplied by the estimated weight to determine the price per animal. The net profits per animal for holding until the slaughter date or selling at live auction were compared. Additional details are provided in Table S7.

Additionally, the DE_PopTK model was applied to evaluate whether low tissue PFOS levels could be obtained without purchasing uncontaminated feed by prioritizing feed expected to have the lowest PFOS concentrations to animals destined for sale or slaughter and timing the feeding of bales from highest to lowest PFOS concentrations prior to sale or slaughter. This scenario would require the ability to identify the field from which each bale was sourced and assumes that feed with the highest PFOS levels on the farm would be fed to breeding stock. Simulations were run to evaluate feed prioritization and timing at two cattle life stages: (1) the first winter of life for cattle to be sold at live auction at 18 months and (2) the second winter of life for cattle raised for slaughter at 24 months. These simulations were constrained by the actual number of hay bales recorded to be harvested from each field and the fact that some feed expected to have higher PFOS levels was needed to meet feed requirements for animals intended for sale or slaughter. Feed selection and timing were based on PFOS soil levels and the expected concentrations in feed, and estimated PFOS muscle levels were compared to PFOS action levels. Additional details are provided in the Supplementary Materials.

## 3. Results

### 3.1. PFAS Measurements in Soil, Water, and Forage

PFOS and PFDA soil concentrations for each farm field are shown in Figure 1 and provided in greater detail in Table S9. Elevated concentrations were observed in fields with known biosolids application, with PFOS soil concentrations ranging from 8.2 to 19.8 ng/g and PFDA concentrations ranging from 65.2 to 111.0 ng/g. PFDA soil concentrations were consistently higher than PFOS, with PFDA concentrations on average 8-fold higher. Low soil PFAS concentrations were measured in pastures and hay fields not licensed for biosolids application, with PFOS soil concentrations ranging from below the method detection limit (MDL) to 1.0 ng/g and PFDA soil concentrations ranging from below the MDL to 2.8 ng/g (PFOS MDL = 0.2 ng/g and PFDA MDL = 0.1 ng/g). Other PFAS detected in the soil are described in Simones et al. [14] (referenced as Farm C fields).

The water sampling results are also shown in Figure 1. Sampling of the farm well water prior to filter installation yielded a PFOS concentration of 1.3 nanograms per liter (ng/L), while PFDA was not detected (PFDA MDL = 0.3 ng/L). Periodic sampling after filters were installed consistently resulted in PFOS and PFDA levels below detection or reporting limits (PFOS MDL = 0.4–0.5 ng/L and RL = 1.7–1.8 ng/L, PFDA MDL = 0.3 ng/L and RL = 1.8 ng/L). PFOS and PFDA were higher in surface water samples collected from a small pond accessible from Field 8 (Figure 1; PFOS = 2.9 ng/L, PFDA = 5.0 ng/L) and a stream accessible from Field 14 (Figure 1; PFOS = 11.2 ng/L, PFDA = 49.8 ng/L). These surface water sampling results are provided for completeness and were not utilized in exposure modeling, as surface water is not expected to be a significant source of drinking water for cattle at this farm.

The PFOS and PFDA concentrations in composite cut hay samples collected during the 2023 growing season are summarized in Table 4 and provided in greater detail in Table S10. The measured PFOS and PFDA levels in cut hay ranged from 0.35 to 15.7 ng/g for PFOS and 0.94 to 24.8 ng/g for PFDA. On average, PFDA was present in hay at concentrations 3-fold higher than those of PFOS.

### 3.2. Biomonitoring of PFAS in Cattle Tissue

The animal biomonitoring results are provided in Tables S11–S16. The PFOS and PFDA concentrations in the initial frozen beef sample collected in 2021 were 2.87 and 4.29 ng/g, respectively. Serial sampling of cows conducted in 2022, following a switch to purchased hay from locations with no known biosolids application history, showed steadily decreasing serum and muscle results over the 80-day monitoring period prior to slaughter. The average PFOS and PFDA serum levels at the first timepoint were 24.6 ± 2.69 ng/mL and 35.8 ± 5.41 ng/mL, respectively, and had dropped to 15.5 ± 1.29 ng/mL and 17.5 ± 2.15 ng/mL by the end of the monitoring period. Similarly, muscle levels dropped from mean initial concentrations of 2.25 ± 0.29 ng/g for PFOS and 3.30 ± 0.61 ng/g for PFDA, to 0.66 ± 0.21 ng/g for PFOS and 0.80 ± 0.38 ng/g for PFDA.

Antemortem serum samples collected from yearling cattle in 2023 following six months of contaminated feed followed by 49 days on pasture contained 12.2 ± 2.24 ng/mL PFOS and 14.6 ± 3.93 ng/mL PFDA. These cattle spent an additional 82 days on pasture before the next serum sample collection, with unexpected access to contaminated supplemental hay in the final 2–3 days prior to sampling. The PFOS and PFDA levels measured in these serum samples were 13.4 ± 2.47 ng/mL and 16.1 ± 2.56 ng/mL, respectively.

Antemortem serum sampling conducted in 2024 with yearlings after six months of exposure to contaminated feed revealed mean serum levels of 89.1 ± 7.97 ng/mL for PFOS and 202 ± 18.6 ng/mL for PFDA. Repeat serum samples collected from the same animals after four months of depuration on pasture showed reduced mean PFOS and PFDA concentrations of 18.8 ± 4.75 ng/mL and 20.3 ± 6.65 ng/mL, respectively.

Paired muscle and serum data collected from four Maine beef and dairy farms, including the study site, are provided in Table S16. At one of these farms, PFAS was only measured in plasma. These data were adjusted to estimated serum concentrations using serum/plasma ratios of 1.060 for PFOS and 1.047 for PFDA from data provided in Table S15. Paired PFOS muscle and serum data were collated with datasets previously included in a PFOS muscle partitioning meta-analysis [30]. The expanded dataset resulted in an updated meta-mean PFOS PM of 0.08 ± 0.02. Additionally, a meta-analysis was conducted for PFDA muscle partitioning, resulting in a meta-mean PFDA PM of 0.08 ± 0.02.

### 3.3. PFAS Exposure

Estimated or measured PFOS and PFDA concentrations in stored feed, pasture forage, soil, and water are summarized for each exposure scenario in Tables S17–S20. For first-cut hay, estimated PFOS concentrations ranged from 1.2 to 1.3 ng/g, and PFDA concentrations ranged from 2.1 to 2.3 ng/g. For second-cut hay, harvested only in 2020 and 2021 from a subset of fields, estimated PFOS and PFDA concentrations ranged from 1.2 to 2.8 ng/g and 1.6 to 3.2 ng/g, respectively. Weighted-average feed concentrations for the 2024 exposure scenario, calculated using PFAS measured in cut hay, were calculated to be 5.9 ng/g for PFOS and 11.7 ng/g for PFDA. Weighted-average feed concentrations based on PFAS measured in hay were several-fold higher than concentrations in feed estimated from soil PFAS levels and plant transfer factors (Table S20). Excluding Field 8, concentrations in pasture forage were estimated to be under 0.1 ng/g for both PFOS and PFDA. Field 8, with a history of biosolids application and used both to grow hay and for fall grazing, had estimated levels of 3.8 ng/g for PFOS and 4.9 ng/g for PFDA.

There was no seasonal variation in water exposure. Based on sampling prior to water treatment and assuming that the well water provided in outdoor and barn watering troughs was the only source of drinking water, PFOS in water was estimated to be 1.3 ng/L until the date water filtration began—after which PFOS was assumed to be 0 ng/L. As PFDA was not detected in well water prior to filtration, PFDA was assumed to be constant at 0 ng/L.

### 3.4. Model Evaluation

**Figure 2:**
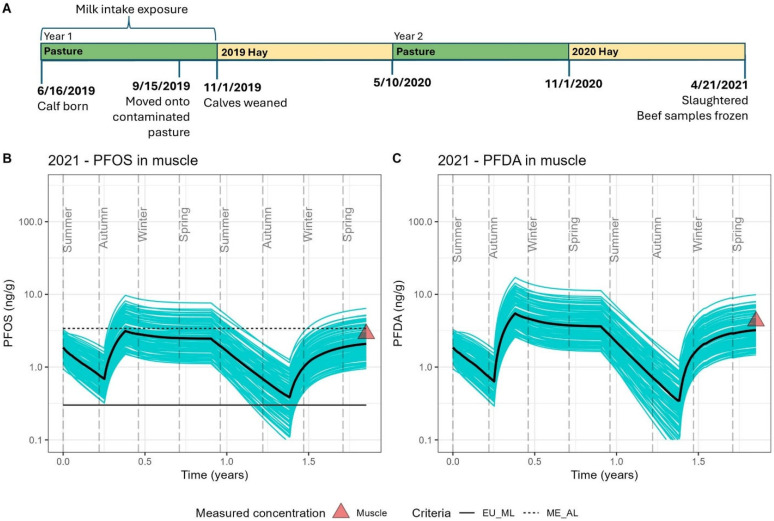
The 2021 exposure scenario. (**A**) Timeline detailing the exposure history of a two-year-old beef cow slaughtered with frozen beef sampled in 2021. (**B**) Estimated PFOS concentrations in muscle over the lifespan of this animal. (**C**) Estimated PFDA concentrations in muscle over the lifespan of this animal. DE_PopTK-model-estimated concentrations over the two-year period are provided as 100 individual stochastic simulations represented by blue lines, with the median estimate presented as a black line. Measured PFAS concentrations in the frozen beef sample are presented as red triangles. Modeled and measured PFOS concentrations in muscle are compared to current health criteria, with the European Union (EU) maximum limit (0.3 ng/g) shown as a solid line and the Maine action level (3.4 ng/g) shown as a dashed line.

**Figure 3:**
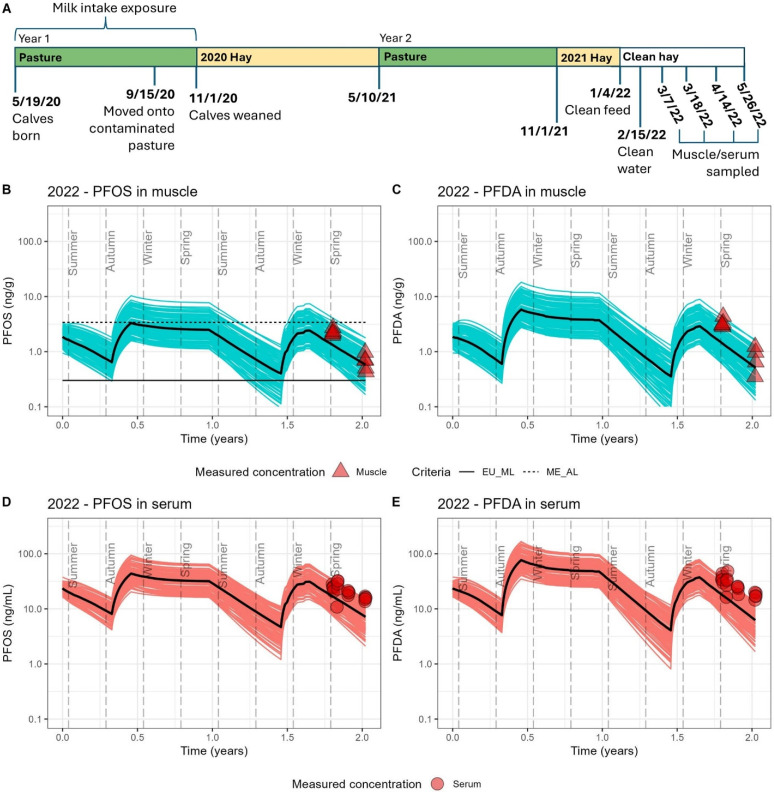
The 2022 exposure scenario. (**A**) Timeline detailing the exposure history of 5 two-year-old animals sampled in 2022. (**B**) Estimated PFOS concentrations in muscle. (**C**) Estimated PFDA concentrations in muscle. (**D**) Estimated PFOS concentrations in serum. (**E**) Estimated PFDA concentrations in serum. Cattle were sampled at four timepoints during a period of depuration, with serum sampled at each timepoint and muscle sampled at the initial and final timepoints. DE_PopTK-model-estimated concentrations over the two-year period are provided as 100 individual stochastic simulations represented by blue lines for muscle and red lines for serum, with median estimates presented as a black line. Measured PFAS concentrations in muscle and serum are presented as red shapes. Modeled and measured PFOS in muscle are compared to current health criteria, with the European Union (EU) maximum limit (0.3 ng/g) shown as a solid line and the Maine action level (3.4 ng/g) shown as a dashed line.

**Figure 4:**
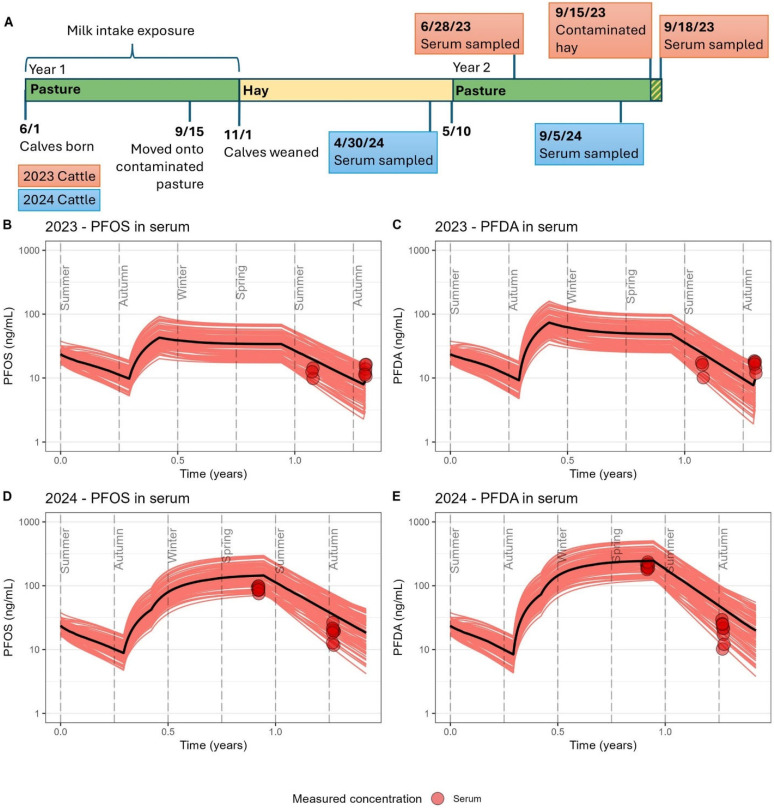
The 2023 and 2024 exposure scenarios. (**A**) Timeline detailing exposure histories for two groups of cattle sampled at around 12 to 18 months of age, with events specific to the 2023 cattle group (*n* = 6) shown in red and events specific to the 2024 cattle group (*n* = 8) shown in blue. (**B**) Estimated PFOS concentrations in serum for the 2023 cattle. (**C**) Estimated PFDA concentrations in serum for the 2023 cattle. (**D**) Estimated PFOS concentrations in serum for the 2024 cattle. (**E**) Estimated PFDA concentrations in serum for the 2024 cattle. DE_PopTK-model-estimated concentrations over time are provided as 100 individual stochastic simulations represented by red lines with the median estimate presented as a black line. Measured PFAS concentrations in serum are presented as red circles.

The model results for each exposure scenario are presented in Figure 2, Figure 3 and Figure 4, and comparisons of mean model predictions to mean measured concentrations of PFOS and PFDA in muscle and serum are summarized in Table 5. Across the four scenarios, a seasonal pattern of accumulation and depuration was observed with both PFOS and PFDA, coinciding with cattle cycling through summers grazing on pasture fields with low soil contamination and winters consuming feed sourced from fields with higher levels of soil contamination. Modeled serum and muscle levels demonstrated sensitivity to other documented exposure events over the course of a lifetime for cattle at the study farm, such as the transition from nursing to forage intake (Figure 2, Figure 3 and Figure 4), rotation onto a contaminated field in the fall for grazing in the first year of life (Figure 2, Figure 3 and Figure 4), the interruption of an accumulation period with the introduction of purchased feed presumed to be low in PFAS (Figure 3), the brief feeding of contaminated supplemental hay prior to serum sampling during a typical depuration period on pasture (Figure 4B,C), and the much higher serum levels from consuming the 2023 hay crop with unusually high PFAS contamination (Figure 4D,E). Good agreement was achieved for both PFOS and PFDA between the model estimates and measured biomonitoring data. Across all scenarios, mean model estimates were within a factor of 2 of the mean measured concentrations in muscle and serum (Table 5). The percentage error between modeled mean predictions of serum and muscle tissue as compared to measured tissue levels ranged from −19% to 68% for PFOS and −16 to 73% for PFDA, with a mean absolute percentage error of 43% for both PFOS and PFDA. PFOS and PFDA muscle and serum measurements were largely captured within prediction bands representing results from 100 stochastic simulations. The model performed similarly well in predicting tissue levels following a period of accumulation (Figure 2 and Figure 4D,E) and during periods of depuration (Figure 3 and Figure 4B,C). Some differences in model performance between scenarios were observed. The model demonstrated a tendency to underpredict PFOS and PFDA concentrations in muscle and serum for scenarios in which cattle reached an age of at least 20 months and were sampled during their second winter on contaminated feed. The model tended to overpredict serum levels in scenarios simulating younger cattle (12 to 18 months old) sampled after their first winter of exposure.

Muscle samples collected in 2021 and 2022 were below Maine’s current PFOS beef tissue action level of 3.4 ng/g, although approximately 15% of 2021 model simulations indicated the potential for tissue levels to exceed this action level at time of slaughter given the potential for variability of feed intake and toxicokinetics (Figure 2) [47]. In the 2022 scenario with depuration on purchased clean feed prior to slaughter, 100% of model simulations fell below Maine’s PFOS beef action level at time of slaughter, with roughly 15% of simulations falling below the EU maximum limit for PFOS in beef of 0.3 ng/g (Figure 3) [48]. The model results also indicate the feasibility for animals to achieve PFOS tissue levels approaching the EU maximum limit following a summer of depuration on pastures with PFOS and PFDA soil levels generally less than 1 ng/g. For the 2021 and 2022 scenarios, 35% of the model simulations fell below the EU maximum limit after approximately six months on these pastures (Figure 2 and Figure 3).

### 3.5. Application as Farm Management Tool

The modeled and measured tissue concentrations indicated that it was feasible to attain muscle levels approaching the lower EU maximum limit after a period of providing clean feed (Figure 3B) or a summer of grazing on pastures with low PFAS soils (Figure 2B). The estimated net revenue the farmer would receive under the current business model of holding until slaughter at 24 months, including the purchase of clean feed prior to slaughter, is USD 2040 per animal. The estimated net revenue from selling 18-month-old cattle in live auction following a summer of grazing is USD 1444 per animal. The price differential between these two options was sensitive to the amount of supplemental grain used for finishing cattle, and grain intake at this farm is around 15–20% of total dry matter intake in the final months prior to slaughter.

Model-predicted PFOS muscle levels for management simulations of prioritized and timed feeding of bales expected to contain the least PFOS are illustrated in Figures S4A and S5A. Model-predicted muscle levels of the sum of PFOS and PFDA resulting from these feeding scenarios are provided in Figures S4B and S5B. Optimizing and timing feed earlier during the first winter of life followed by 6 months of grazing before live auction sale resulted in a mean predicted PFOS concentration in beef muscle of 0.2 ng/g at the time of live auction, with 90% of predictions falling below the EU maximum limit (Figure S4A). Implementing this management strategy in the second winter of life, for the final 6 months prior to slaughter, resulted in a mean predicted PFOS concentration in beef muscle of 0.8 ng/g at slaughter, with 100% of predictions being below Maine’s current beef action level but less than 10% of predictions being below the EU maximum limit (Figure S5A). Including estimated PFDA muscle levels in the above simulations indicated that the optimization of hay feeding based on PFOS levels at this farm also optimized for PFDA-related feed exposures, as illustrated in Figures S4B and S5B.

## 4. Discussion

Previous evaluation of the DE_PopTK model largely considered beef farms impacted by historical use of aqueous film-forming foams and water-dominated exposures [30]. The present work shows that this modeling approach also works well when applied to an exposure setting dominated by a soil and forage crop contamination pathway presumably resulting from the historical application of biosolids. We made modifications to include lactational exposure to calves born on the farm and expanded upon previous work to include parameterization for PFDA. Good agreement was achieved for both PFOS and PFDA between model estimates and biomonitoring data representative of periods of significant accumulation or depuration when tissue PFAS levels were expected to be near maximum levels or minimum levels. These results were replicated over multiple years with different groups of animals on the same farm.

Model performance was closely tied to the application of site-specific data to describe farm exposures, including PFAS soil levels for each field used for stored feed or pasture, recorded hay yields for each field, farmer-reported yields and feeding patterns of first- and second-cut hay, site-specific data for plant uptake of PFAS, and, in one scenario, the use of PFAS levels measured in stored feed. Understanding the seasonal exposure pattern at this farm resulting from contaminated feed sourced from biosolids-amended fields and summer grazing on pastures without a history of biosolids application was integral to model performance. The average PFOS and PFDA concentrations in all stored feedstocks were estimated based on site-specific plant transfer factors and fieldwide average PFAS soil levels weighted by the hay yields recorded for each field. This averaging approach was required due to the lack of specific information on when bales from individual fields were fed out. Despite the several-fold range in soil PFAS concentrations measured in the farm’s hay fields and thus similar differences in PFAS concentrations in feed, this averaging approach appeared to provide reasonable estimates of exposure, as evidenced by the agreement between the modeled and measured muscle and serum levels for three of the four exposure scenarios (Figure 2, Figure 3 and Figure 4, Table 5). Adjusting feed concentrations from second-cut hay using recorded second-cut hay yields, farmer-reported typical feeding patterns, and assuming a 2.7-fold and 2.1-fold increase in second-cut grass uptake of PFOS and PFDA, respectively, resulted in modest improvements in model performance (up to a 23% reduction in percentage error for PFOS and a 7% reduction for PFDA when comparing modeled and measured data). At this farm, second-cut hay was only harvested in two out of the five seasons utilized for exposure modeling and was a relatively small fraction of the total hay yields in those years (6–14% of total crop). Greater yields and the usage of successive cuttings for livestock feed would likely have a greater impact on muscle levels, depending on the rate and timing of feeding relative to slaughter dates.

In the 2024 scenario, the use of fieldwide average soil concentrations and site-specific plant transfer factors resulted in a substantial underprediction of PFAS concentrations in stored feed as compared to concentrations measured in cut hay samples collected in the summer of 2023 and fed to the 2024 cattle group. Modeling feed exposure using site-specific plant transfer factors in this scenario underestimated the measured serum levels by more than 2-fold for PFOS and more than 4-fold for PFDA (Figure S5). The use of the PFAS levels measured in hay samples resulted in good agreement between the modeled and measured serum levels. The reason for the substantial difference between the estimated and measured PFAS levels in hay harvested in the summer of 2023 is not known. Model predictions for 2021, 2022, and 2023 scenarios relying on PFAS feed levels estimated from soils and plant transfer factors agree well with the measured tissue levels (Figure 2, Figure 3 and Figure 4B,C). It is possible that the observed measured plant PFAS levels may have been related to unusually heavy precipitation in central Maine during the summer of 2023. Simones, et al. [14] reported a tendency for higher plant uptake of PFOS this same year relative to prior years for study plots located on another farm in central Maine. Higher than expected PFAS plant levels may also be related to a later than typical harvest, as these samples were collected from fields first cut in late July through September. In prior years, the farmer reported harvesting a second cutting from several fields in August through September. These observations and model findings illustrate the importance of site-specific data to inform exposure modeling and the need to consider the potential for significant variation in PFAS plant uptake related to spatial and temporal factors [14].

Biomonitoring during a period of depuration conducted at this farm provides additional data on the kinetics of PFOS and PFDA depuration in beef tissues. While the duration of serial sampling was not sufficient to provide a robust estimate of half-lives, it is noteworthy that the observed declines in serum PFOS and PFDA levels were consistent with serum half-lives reported by Drew, et al. [29] and shorter than those reported for PFOS by Lupton, et al. [54]. We cannot rule out potential low-level ongoing exposure during our monitoring of depuration while animals were provided with clean feed purchased offsite.

There is increasing concern regarding biosolids applications and health risks for those consuming food commodities sourced from PFAS-contaminated soils. The U.S. EPA recently released a draft risk assessment for PFAS in biosolids that reports human health risks exceeding the U.S. EPA’s acceptable thresholds for some scenarios where biosolids containing 1 ng/g of PFOA or PFOS were applied to farmland. However, this assessment has a more prospective regulatory framework rather than considering decades of historical land spreading [55]. Our findings demonstrate that PFOS soil concentrations ranging from 8 to 20 ng/g, presumably from the historical application of contaminated biosolids, resulted in PFOS beef tissue levels well above the EU’s maximum limit of 0.3 ng/g and approaching Maine’s action level of 3.4 ng/g as well as the USDA’s interim screening level of 4.1 ng/g [47,48,56]. It is noted that the PFDA soil levels on this farm were appreciably higher than those for PFOS (ranging from 65 to 111 ng/g on biosolids applied fields), and similarly, PFDA serum and muscle levels were consistently higher than those for PFOS. Muscle tissue data indicated combined concentrations for PFOS and PFDA in beef ranging from 5.6 to 7.2 ng/g. To our knowledge, at the time of writing, no governmental agency has developed guidance values for PFDA in beef muscle. Based on the U.S. EPA’s recently finalized reference dose for PFDA, it is likely that any limits for PFDA in beef will be at least of similar magnitude to, if not lower than, those for PFOS [57]. PFDA measurements at this farm indicate the potential for elevated levels of PFDA to be present at sites with biosolids-related contamination, as well as the tendency for PFDA to accumulate in cattle. These findings warrant consideration of PFDA in health assessments conducted at biosolids sites, as well as consideration of the combined exposure to both PFOS and PFDA.

Dynamic modeling of beef farms has been previously applied to investigate exposure management measures such as providing treated water or altering the timing of rotation onto contaminated fields [30]. We have further demonstrated the utility of exploring farm management interventions augmented with economic analyses to investigate options of selling live cattle following a summer of depuration or slaughtering to sell meat directly to consumers following the feeding of purchased non-contaminated feed. These analyses indicated that purchasing non-contaminated feed prior to slaughter would be the more profitable option for this farm and allow the farmer to consider whether the difference in profit would be worth the associated added time and labor. It is noted that this is a small-scale farm without significant grain or labor costs. Feed costs are also likely to vary from year to year, which may impact management decisions. We additionally used the model to evaluate a management strategy of tracking bales by field so that feed sourced from the fields with the lowest PFAS levels can be strategically fed to minimize body burden at either slaughter or live auction. These simulations indicated that it is possible for this beef farm to remain viable with relatively modest changes to management practices despite elevated soil PFOS and PFDA levels.

The DE_PopTK model, originally developed by Mikkonen et al. [30] and expanded in this present work, adds to a growing set of tools of varying complexity available for assessing the PFAS exposure of livestock and informing farm management practices on contaminated land [27,58,59]. Soil screening levels, such as those developed by Maine CDC [27] for dairy farming and soon to be extended to beef farming, can be used to identify when PFAS soil levels are of potential concern and warrant further site-specific farm investigation. Online tools like ConTrans, provided by the German Federal Institute for Risk Assessment [59], can be used to estimate tissue levels when exposure is fairly constant or to estimate depuration times based on known or estimated tissue levels. Dynamic exposure models, such as the DE_PopTK described here, require a significant amount of detailed exposure information to leverage their full capabilities, and their use may be reserved for situations warranting the investment of resources to obtain the needed exposure data. This may include situations where a farm needs to adjust management practices to reduce contamination levels in their beef product but the feasibility or specific changes are not immediately clear. In those instances, proper utilization of a dynamic exposure model may be the most promising avenue to achieving farm viability. Additionally, utilization of a dynamic exposure model may be a cost-effective method for a regulatory agency to design long-term sampling plans to ensure product safety. Antemortem blood testing, such as that described by Johnston et al. [56], can be used to monitor estimated beef tissue levels as a check on the various predictive tools and to monitor for potential changes in exposure due to factors such as the temporal variability in plant uptake described here and elsewhere [14]. Collectively, these tools provide a way to develop interim strategies for managing PFAS contamination on cattle farms as we await the development of effective interventions such as feed additives to reduce absorption or enhance elimination of PFAS in cattle and soil amendments to reduce plant uptake of PFAS from soil [60,61].

## 5. Conclusions

In this study, we apply and extend the Mikkonen, et al. [30] DE_PopTK model to a farm setting where the primary PFAS exposure pathway for livestock is contaminated soil resulting in contaminated grass-based forages. PFAS-contaminated soils were presumably the consequence of decades of historical land application of biosolids. We extended the model for PFOS to additionally estimate PFDA exposure and the resulting tissue levels. We collected and made use of extensive farm-specific data, including farm management practices, spatial variation of PFOS and PFDA levels in soil and water, plant uptake, and seasonal and annual variability in daily exposure estimates based on water, feed, and soil intake. We documented a highly dynamic exposure pattern as cattle accumulated PFAS while consuming stored feed grown on contaminated land and depurated while grazing on non-contaminated pastures. The model-estimated PFOS and PFDA levels in serum and muscle were in good agreement with the biomonitoring data collected from four different groups of cattle sampled over a four-year period representing periods of accumulation and depuration, animals of different ages, and seasonal and annual changes in environmental conditions. We observed substantial variation in PFOS and PFDA contents in grass-based forages in some years for reasons that are not well understood but could include the timing of harvest and seasonal precipitation. Collectively, our findings demonstrate that understanding variation in farm exposures and collecting site-specific data were integral to model performance. Future work is needed to better understand the factors that contribute to within-season and year-to-year variation in the plant uptake of PFAS from contaminated soil. We have demonstrated that the DE_PopTK model can be a useful tool to simulate farm management strategies as an aid to identifying practices that can result in lower PFOS and PFDA levels in beef to maintain farm viability despite elevated PFAS soil levels. Our work adds to a growing set of tools of varying complexity available for assessing PFAS exposure of livestock and informing farm management practices on contaminated land.

## Figures and Tables

**Table 1:** Animal biomonitoring summary.

Location	Date(s)	*n* ^a^	Cattle Age (Years)	Cattle Type	Tissues Sampled	Data Application
Study site	7 December 2021	1	2	Beef	Muscle	Exposure model: 2021 scenario
Study site	7 March, 18 March, 14 April, and 26 May 2022	5	2	Beef	Muscle, Serum, Plasma	Exposure model: 2022 scenario
Study site	28 June and 18 September 2023	6	1.5	Beef	Serum	Exposure model: 2023 scenario
Study site	30 April and 5 September 2024	8	1.5	Beef	Serum	Exposure model: 2024 scenario
Offsite	11 October 2022	5	10	Beef	Muscle, Serum, Plasma	Model parameterization: muscle partition coefficient
Offsite	25 November 2020 and 3 September 2021	19	NR ^b^	Beef, Dairy	Muscle, Plasma	Model parameterization: muscle partition coefficient
Offsite	13 October 2022	3	2-4	Beef	Muscle, Serum	Model parameterization: muscle partition coefficient
Offsite	4 November 2022	12	NR ^b^	Beef, Dairy	Serum, Plasma	Model parameterization: serum/plasma ratio

^a^ Number of animals sampled. ^b^ Not recorded. All samples were obtained from adult animals.

**Table 2:** Description of cattle group exposure scenarios.

Exposure Scenario	Exposure History
2021	This animal spent two summers on pasture and two winters on stored feed prior to slaughter in April 2021 at 2 years of age. Second-cut hay was fed during its second winter only. The primary drinking water source was untreated well water.
2022	Cattle spent their first and second summers on pasture and first winter on stored feed. For the second winter, cattle were fed stored feed for 2 months, followed by an intervention in which clean feed was purchased to allow cattle to depurate for 4 months (grab sampling of purchased feed confirmed PFOS and PFDA were not present above detection limits). First- and second-cut hay was used as feed in both winters. Cattle were provided with untreated well water until February 2022, after which all drinking water was treated. They were slaughtered at 2 years of age.
2023	Cattle spent two summers on pasture and one winter on stored feed. No second-cut hay was fed during this time. During their second summer on pasture, access to contaminated supplemental hay was provided 2–3 days prior to the second serum sampling. Their primary drinking water was treated well water.
2024	Cattle spent two summers on pasture and one winter on stored feed. No second-cut hay was fed during this time. Their primary drinking water was treated well water.

**Table 3:** Toxicokinetic model parameters.

Analyte	Parameter	Units	Mean	SD	Reference
PFOS	DT50	days	74.1	13.4	Drew et al. [29]
	PM	unitless	0.08	0.02	Mikkonen et al. [30], Maine farm data
	Pmilk	unitless	0.015	0.004	Mikkonen et al. [30]
	Vd	L/kg	0.085	0.01	Mikkonen et al. [30]
PFDA	DT50	days	60.4	10.4	Drew et al. [29]
	PM	unitless	0.08	0.02	Maine farm data
	Pmilk	unitless	0.015 ^a^	0.004	Mikkonen et al. [30]
	Vd	L/kg	0.085 ^a^	0.01	Mikkonen et al. [30]

^a^ PFOS parameter value was applied as PFDA data were not available. PFDA toxicokinetics in beef cattle are assumed to be similar to PFOS.

**Table 4:** PFOS and PFDA concentrations in cut hay samples used in the 2024 exposure scenario.

Hay Field # ^a^ (Acres)		PFOS		PFDA	
	*n* ^b^	Mean ± SD	Range	Mean ± SD	Range
2 (15)	3	3.44 ± 1.47	1.83–4.71	18.64 ± 4.72	14.93–23.96
3 (1)	1	6.89		24.85	
6 (8)	3	5.11 ± 2.59	2.13–6.82	18.47 ± 1.12	17.21–19.38
7 (13)	8	8.47 ± 3.88	3.33–15.68	11.87 ± 3.18	8.07–16.09
8 (14)	5	7.11 ± 3.82	0.35–9.76	14.21 ± 7.70	0.94–20.84

^a^ Field numbers correspond to Figure 1A. ^b^ Number of composite sample areas within each field. Sample areas were selected to be representative of field size and area cut for hay.

**Table 5:** Measured PFOS and PFDA concentrations in muscle (ng/g) and serum (ng/mL) compared to model estimates for each sample group exposure scenario.

Sample Group	*n*	PFAS	Tissue	Measured ^a^		Modeled ^b^		
				Mean ± SD	Range	Mean ± SD	Range	% Error ^c^
2021	1	PFOS	Muscle	2.87		2.33 ± 1.12	0.95–6.40	−19%
		PFDA	Muscle	4.29		3.61± 1.73	1.47–9.90	−16%
2022	5	PFOS	Muscle	2.25 ± 0.29	1.95–2.71	1.48 ± 0.73	0.57–4.09	−34%
		PFOS	Serum	24.6 ± 2.69	20.7–28.2	17.6 ± 6.53	7.49–34.3	−29%
		PFDA	Muscle	3.30 ± 0.61	2.82–4.35	1.56 ± 0.78	0.58–4.32	−53%
		PFDA	Serum	35.8 ± 5.41	29.6–43.9	18.6 ± 6.97	7.62–36.3	−48%
2023	6	PFOS	Serum	12.2 ± 2.24	9.84–14.3	20.3 ± 7.78	7.91–39.9	67%
		PFDA	Serum	14.6 ± 3.93	10.1–17.5	25.2 ± 9.87	9.25–49.5	73%
2024	8	PFOS	Serum	89.1 ± 7.97	74.9–98.8	150 ± 52.9	70.3–295	68%
		PFDA	Serum	202 ± 18.6	179–235	256 ± 90.5	121–502	27%

^a^ Concentrations measured at initial sampling timepoints are provided. ^b^ Model estimates corresponding to the time of initial sampling, presented as mean estimates, standard deviations, and ranges of 100 stochastic simulations. ^c^ Percentage errors are calculated comparing DE_PopTK model mean estimates to mean measured PFAS in muscle or serum.

## Data Availability

Much of the data generated in this work are provided in tables in the Supplementary Materials. Additional data may be available upon request but will need to comply with any restrictions due to requested privacy requirements for the farm. Computer code is provided as files in the Supplementary Materials.

## Supplementary Materials

The full R code, sample model input files, and the following supporting information can be downloaded at: https://www.mdpi.com/article/10.3390/toxics13070541/s1, Figure S1: Map of soil sampling methods; Table S1: Interlaboratory comparison; Table S2: Hay records by field; Table S3: Plant transfer factors for first and second cut hay; Table S4: Dry hay and wrapped baleage records; Table S5: Grazing rotation schedule in cattle first year of life; Table S6: Grazing rotation schedule in cattle second year of life; Table S7: Economic analysis summary; Table S8: Average number of bales harvested by field; Table S9: PFAS soil concentrations by field; Table S10: PFAS concentrations in cut hay; Table S11: 2021 PFAS measured in muscle; Table S12: 2022 PFAS measured in muscle, serum, and plasma; Table S13: 2023 PFAS measured in serum; Table S14: 2024 PFAS measured in serum; Table S15: PFAS in paired serum and plasma samples; Table S16: PFAS in paired serum and muscle samples; Figure S2: Forest plots depicting meta-analyses of muscle:serum partition coefficients; Table S17: 2021 Exposure model inputs; Table S18: 2022 Exposure model inputs; Table S19: 2023 Exposure model inputs; Table S20: 2024 Exposure model inputs; Figure S3: PFAS Vd sensitivity analysis; Table S21: Timed feeding simulation exposure inputs; Figure S4: Simulation of timed feeding in first year of life; Figure S5: Simulation of timed feeding in second year of life; Figure S6: Alternative model run for 2024 exposure scenario using soil-to-plant transfer factors. References [62,63,64,65] are cited in Supplementary Materials.
