# Supplementary material for: Application of a Dynamic Exposure Population Toxicokinetic Model for Perfluorooctane Sulfonic Acid (PFOS) and Extension to Perfluorodecanoic Acid (PFDA) at a North American Beef Cattle Farm with a History of Biosolids Land Application
Source: Toxics. 2025 Jun 27;13(7):541. doi: 10.3390/toxics13070541 (PMC12300002; doi:10.3390/toxics13070541)
Supplement: Supplementary file 1 [file toxics-13-00541-s001.zip › toxics-3667938-supplementary.pdf]

Supplementary Materials

Application of a dynamic exposure population toxicokinetic model for perfluorooctane sulfonic acid (PFOS) and extension to perfluorodecanoic acid (PFDA) at a North American beef cattle farm with a history of biosolids land application

Authors and Affiliations:

Barbara A. Astmann[1†], Antti T. Mikkonen[2†], Thomas L. Simones[1], Meghan Flanagan[3], Duncan Pfaehler[3], Ivan Lenov[4], Andrew E. Smith[1] *

[1] Maine Center for Disease Control and Prevention, Augusta, Maine 04333, United States
[2] Environment Protection Authority (EPA) Victoria, Centre for Applied Sciences, Macleod, Victoria 3085 Australia
[3] Department of Agriculture, Conservation and Forestry, Augusta, Maine 04333, United States
[4] U.S. Department of Agriculture Food Safety and Inspection Service, Saint Louis, Missouri, 63120
*Corresponding author e-mail address: andy.e.smith@maine.gov

Soil Sampling Methods

Three methods of obtaining fieldwide composite soil samples were utilized at the study farm. Variations in soil sampling methods throughout the farm are described below and depicted in Figure S1. As part of an initial site investigation for regulatory purposes, the farm's four pasture fields were sampled using 10-part composite sampling in a dispersed pattern intended to be generally representative of each field. Following this initial sampling, 15- and 30-part composite samples were collected within each of the farm's three primary hay fields using a gridded sampling method. Gridded field overlay maps were generated with 15- and 30-grid cells of equal shape and size within each field and coupled with handheld GPS devices to navigate to the centroid of each grid cell. Triplicate soil samples were collected from each 15 and 30 grid cell; one replicate at each cell centroid and two replicates offset approximately 3 meters from each centroid. Comparison of results obtained from 15-grid and 30-grid methods showed limited variation among replicates and modest differences between 15-part and 30-part grid sampling (relative percent differences ranging from 7% to 36% for PFOS and 11% to 44% for PFDA). The farm's remaining hay fields were all sampled using the 15-part gridded sampling methodology with only a single sample collected at the centroid of each grid cell.

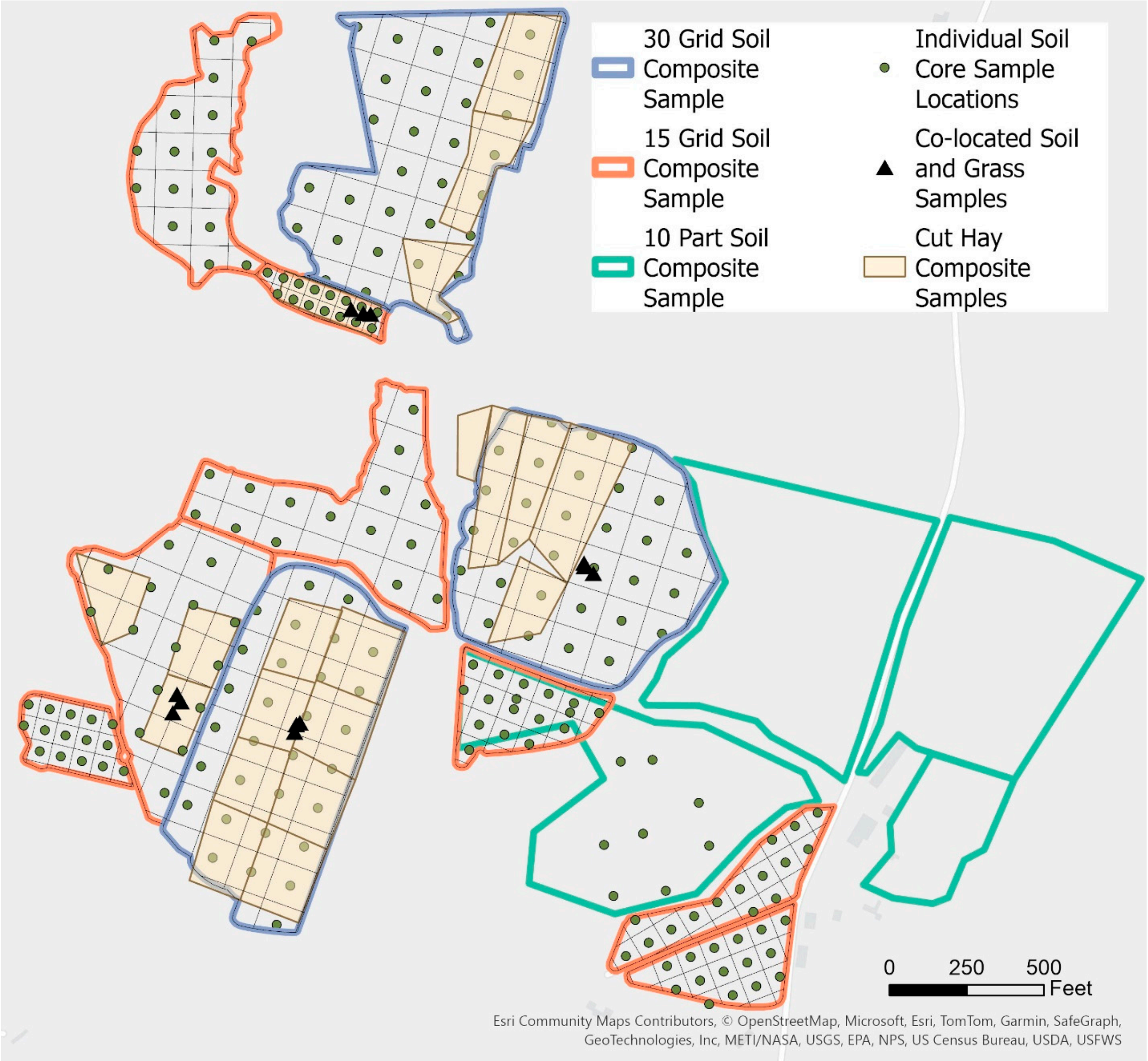

Figure S1. Map detailing which fields were sampled using 10-part, 15-grid, or 30-grid soil composite sampling methodologies. Individual soil core sampling locations are shown within each field, with the exception of three pastures sampled using the 10-part composite method in which individual sample locations were not recorded. Co-located soil and grass samples, and composite cut hay sampling locations are also shown.

Study Farm Biomonitoring

In December 2021, a freezer beef sample from a single animal that had been slaughtered in April 2021 at the typical age of two-years was obtained as part of the initial investigation of PFAS contamination at this farm. In the spring of 2022, a depuration study was initiated to monitor tissue and serum levels in animals provided uncontaminated feed prior to slaughter for meat. Live muscle biopsies were performed on a group of five two-year old cattle on March 7, 2022, two months after being provided feed purchased off-site and sourced from land without any known biosolids application history. Three grab samples collected from purchased feed were not found to contain PFOS or PFDA above limits of detection (0.15 ng/g wet weight). Three months later, muscle samples were collected on the same animals at slaughter. Serum samples were collected from these same five cattle on March 7, March 18, April 14, and at slaughter on May 26, 2022. Tissue and serum collection methods are described in the manuscript. The following year, live serum sampling was conducted on an additional group of six 13-month-old cattle on June 28, 2023, after about 1.5 months of grazing on pasture. Serum samples were collected from this group again on September 18, 2023, while animals were still on pasture but had just been fed contaminated supplemental feed. Live serum sampling was conducted on an additional eight 11-month-old cattle on April 30, 2024, after 6 months of consuming contaminated stored feed. Serum was collected from these cattle again on September 5, 2024, after about 4 months of grazing on pasture.

Additional Off-site Biomonitoring

Paired muscle and serum and/or plasma samples were collected at three additional Maine beef and dairy farms to both assess tissue levels prior to slaughter and as part of an effort to develop a muscle serum partitioning dataset for PFAS to be used in making predictions of muscle levels based on measured serum levels. At two of these beef farms, live animal sampling of serum samples and muscle biopsies were collected from a total of eight cattle ranging from 2-10 years of age. At the third farm, paired blood and muscle tissue samples were obtained shortly after animals were humanely euthanized under a herd depopulation in response to PFAS contamination. These depopulation events included both a beef cattle herd and a dairy cattle herd, though the dairy herd was a mixed herd of cows, calves, and bulls and not part of an active dairy operation. Though some of these animals would have been lactating at the time of sample collection, they were not being milked in a production environment and assumed to be producing less milk than typical dairy animals. Thus, these dairy animals were pooled with beef cattle in the muscle serum partitioning dataset. Samples from calves were not included.

Laboratory Methods for PFAS Analyses

Water and soil samples were sent to Pace (Westborough, MA, USA) for targeted analysis of 28 PFAS using their laboratory-defined isotope dilution method based on EPA 537.1 modified method (method summary provided in Simones, *et al.* [1] Supporting Information). PFAS measured in water samples were reported in ng/L, and PFAS measured in soil samples were reported on a ng/g dry weight (dw) basis.

Plant tissue samples were analyzed by the U.S. Food and Drug Administration (FDA) laboratory (Center for Food Safety and Applied Nutrition, College Park MD). Samples were ground using a Cryo-blade cryogenic grinder from Cole Palmer (Vernon Hills, IL, USA), and analyzed in duplicate using an isotope dilution method developed for FDA's total diet study survey analysis (C-010.02) [2,3]. PFAS measured in

plant samples were reported on a ng/g wet weight (ww) basis. FDA did not provide percent moisture data, so an average percent moisture of 15% measured in 4 grab dry hay samples collected from the study farm was used to convert PFAS concentrations to a dry weight basis.

The 2021 and 2022 muscle tissue samples and 2023 serum samples from the study farm were sent to Battelle Laboratories (Norwell, MA, USA) to be analyzed using their DoD QSM 5.3 Table B-15 isotope dilution method for targeted analysis of 28 PFAS (method summary provided in Simones, et al. [1] Supporting Information). The 2024 serum samples from the study farm were also sent to Battelle and analyzed using EPA method 1633 [4]. Serum and plasma samples collected from the study farm in 2022, as well as paired muscle tissue, serum, and plasma samples collected from additional farms were sent to the United States Department of Agriculture (USDA) Food Safety and Inspection Service (FSIS) Midwestern Laboratory (St. Louis, MO) to be analyzed using FSIS Method CLG-PFAS 2.03 [5,6]. Briefly, tissues were homogenized and a 0.5 gram portion was aliquoted into extraction polypropylene tubes. The samples were fortified with internal standards and then diluted with methanol to a final volume of 2.5 mL. Samples were agitated and proceed through extraction that incorporates a protein precipitation step. Samples were aliquoted into polypropylene autosampler vials and injected onto an LC equipped with a C8 column that is interfaced an MS/MS. The identity of each analyte is confirmed by comparing the measured spectra and retention times against those of quality control standards analyzed concurrently in the same batch, under identical conditions, ensuring accuracy and integrity. Concentrations are determined using a calibration curve, using internal standard-normalized responses. The method uses C13- labeled PFAS standards as internal standards, using each labeled compound to normalize the signal of more than one PFAS analyte. PFAS in muscle was reported on a ng/g ww basis. Serum and plasma samples were reported in units of ng/mL.

Interlaboratory comparisons were performed to monitor the consistency of PFAS results obtained from the several commercial and governmental laboratories used for this study. Prior work found good agreement between PFAS results for plant tissue analyzed by FDA as compared to Battelle using their DoD QSM method with a mean relative percent difference (RPD) for PFOS of less than 26% between laboratories [1]. A smaller set of samples with PFDA detected had a mean RPD of 6% [1]. Good agreement was also found for deer and turkey muscle tissue analyzed by the FDA as compared to Battelle using either their DoD QSM method or EPA Method 1633, with a mean RPD of 12% for PFOS [7]. More recently, good agreement was found for beef muscle tissue samples from 5 animals analyzed by FDA and USDA FSIS, with similar mean RPD observed between laboratories of 23% for PFOS and 22% for PFDA (Table S1).

Table S1. Interlaboratory comparison data for PFOS and PFDA measured in 5 beef muscle samples analyzed at FDA and USDA FSIS laboratories.

	**PFOS in Beef Muscle (ng/g)**		**PFDA in Beef Muscle (ng/g)**	
**Animal #**	**FDA**	**USDA**	**FDA**	**USDA**
1	3.1	2.5	2.3	1.9
2	1.5	1.9	1.3	1.6
3	3.8	3.1	2.7	2.2
4	2.4	3.0	1.5	2.1
5	2.4	1.8	1.3	1.1

Exposure Model Methods

Stored Feed Exposures

PFAS concentrations in stored feed were estimated based on soil concentrations measured in fields used to source feed, annual farm records for total hay bales sourced from each field, and site-specific soil-to-plant transfer factors. A weighted average farm-wide soil PFAS concentration was calculated for each year, based on fieldwide composite soil PFAS concentrations in each field weighted by the fraction of total hay crop recorded to be cut from each field relative to the total hay crop during each growing season (Table S2). In years when second-cut hay was also obtained, weighted average PFAS concentrations in soil were calculated separately for first- and second-cut feed, specific to fields sourced for each cutting (Table S2). Farm hay records indicated that second cuttings occurred in years 2020-2021 (relevant to 2021 and 2022 exposure scenarios), as well as total second-cut bales cut from each field.

Table S2. Fractions of total hay crop sourced from each field for each growing season

<table>
<tr><th>Crop</th><th>2019
1st Cut</th><th>2020
1st Cut</th><th>2020
2nd Cut</th><th>2021
1st Cut</th><th>2021
2nd Cut</th><th>2022
1st Cut</th><th>2023
1st Cut</th></tr>
<tr><td>Total Bales</td><td>369</td><td>233</td><td>16</td><td>247</td><td>41</td><td>204</td><td>162</td></tr>
<tr><td>Hay Field</td><td></td><td></td><td></td><td></td><td></td><td></td><td></td></tr>
<tr><td>2</td><td>0.12</td><td>0.09</td><td>0</td><td rowspan="2">0.19 [a]</td><td>0</td><td>0.06</td><td>0.06</td></tr>
<tr><td>3</td><td rowspan="2">0.11 [a]</td><td>0.03</td><td>0</td><td>0</td><td>0.05</td><td>0</td></tr>
<tr><td>1</td><td>0.11</td><td>0</td><td>0</td><td>0</td><td>0</td><td>0</td></tr>
<tr><td>4</td><td>0.11</td><td>0.10</td><td>0</td><td>0</td><td>0</td><td>0.10</td><td>0.10</td></tr>
<tr><td>5</td><td>0</td><td>0</td><td>0</td><td>0</td><td>0</td><td>0</td><td>0</td></tr>
<tr><td>6</td><td rowspan="2">0.34 [a]</td><td rowspan="2">0.37 [a]</td><td>0</td><td rowspan="2">0.25 [a]</td><td>0</td><td rowspan="2">0.44 [a]</td><td>0.10</td></tr>
<tr><td>7</td><td>0</td><td>0.27</td><td>0.33</td></tr>
<tr><td>8</td><td>0.24</td><td>0.28</td><td>0.31</td><td>0.55</td><td>0.37</td><td>0.26</td><td>0.30</td></tr>
<tr><td>9</td><td>0.04</td><td>0</td><td>0</td><td>0</td><td>0.17</td><td>0.04</td><td>0.04</td></tr>
<tr><td>10</td><td>0.02</td><td>0</td><td rowspan="2">0.69 [a]</td><td>0</td><td>0.20</td><td rowspan="2">0.03 [a]</td><td>0.02</td></tr>
<tr><td>11</td><td>0.02</td><td>0.02</td><td>0.01</td><td>0</td><td>0.04</td></tr>
</table>

[a] Number of hay bales were recorded that year as a mix from one or more fields. To calculate overall farm-wide weighted average soil concentrations for each year, first an average soil concentration weighted by acreage was calculated for combined fields. This average soil concentration was then multiplied by the fraction of total feed sourced from the combined fields.

Site-specific soil-to-plant transfer factors were used to estimate PFAS concentrations in feed given weighted average PFAS concentrations in soil. Co-located soil and plant sampling to obtain site-specific plant uptake data was collected in triplicate from four hay fields onsite earlier in the season (June through early July) and is expected to be representative of a first cutting [1]. Given limited and non-significant variation between averages of co-located sampling in each field (0.081 – 0.10 for PFOS and 0.021 – 0.033 for PFDA), means were used to represent single farm-wide averages for plant uptake in first-cut grasses [1]. Farm-wide mean plant transfer factors of 0.094 ± 0.009 and 0.026 ± 0.005 ng/g dw plant/ng/g dw soil were computed for PFOS and PFDA respectively.

Because site-specific data were not available to quantify plant uptake in second-cut grasses, data from the Simones, et al. [1] study reporting differences in uptake between first and second-cut grasses at another Maine farm were used to adjust first-cut site-specific plant transfer factors to estimate increased uptake in second-cut hay. Ratios of PFOS and PFDA plant transfer factors for second-cut hay to first-cut hay were calculated for each study plot (Table S3) [1]. PFOS was measured in first and second-cut hay during two years of the study. The mean second-cut to first-cut transfer factor ratio for each plot, averaged over the two years, was calculated to be 2.7 ± 0.4 (SEM). PFDA was only detected in first and second-cut hay in two out of four study plots during one year of the study, with a mean of 2.1 ± 0.6 (SEM). These ratios were multiplied by the farm-specific plant transfer factors to yield estimated second-cut plant transfer factors of 0.254 for PFOS and 0.055 for PFDA.

Table S3. Mean PFAS and PFDA plant transfer factors in each plot from Simones, et al. [1] plant uptake study at another farm in Maine. Mean ratios of second-cut to first-cut plant transfer factors were calculated and used to adjust the farm-specific plant transfer factor to estimate second-cut plant uptake.

**PFAS**	**Study Plot**	**Year 2**			**Year 3**		
		**1st Cut**	**2nd Cut**	**2nd:1st Cut Ratio**	**1st Cut**	**2nd Cut**	**2nd:1st Cut Ratio**
PFOS	A-1	0.021	0.077	3.7	0.034	0.125	3.7
PFOS	A-2	0.038	0.144	3.8	0.088	ND	-
PFOS	A-3	0.339	0.332	1.0	0.237	0.581	2.5
PFOS	A-4	0.208	0.356	1.7	0.402	0.719	1.8
PFDA	A-1	ND	ND	-	ND	ND	-
PFDA	A-2	ND	ND	-	ND	ND	-
PFDA	A-3	0.027	ND	-	0.043	0.113	2.6
PFDA	A-4	ND	ND	-	0.033	0.051	1.5

Farmer-reported information was used to estimate the timing of feeding first-cut and second-cut feed throughout the duration that cattle consume stored feed. Cut hay is preserved as either dry hay bales or hay silage in wrapped bales (both referred to as stored feed), with total dry bales and wrapped bales harvested in 2020 and 2021 shown in Table S4. The farmer typically feeds 1-2 bales a week to cattle raised for beef, and dry hay and wrapped bales are alternated at a ratio of 2:1. Due to second cuttings occurring late in the summer and the way dry bales are stacked indoors in a barn, second-cut hay would likely be the first of the dry bales selected to be fed as it is the last crop to be placed in the barn. Wrapped bales are stored outside in rows, and there was no available farm information to indicate any order or timing of feeding wrapped bales.

Table S4. Dry hay and wrapped baleage totals during years when farm records indicated first and second cuttings

	1st Cut			2nd Cut		
Year	Total Bales	Dry Hay Bales	Wrapped Bales	Total Bales	Dry Hay Bales	Wrapped Bales
2020	233	186	47	16	0	16
2021	247	191	56	41	11	30

The following assumptions were made to estimate feeding rates of first and second-cut hay. Bales were fed at a rate of 1 bale every 5 days, and both breeding stock and cattle raised for beef were fed at the same rate. Bales were alternated at a ratio of 2:1 dry hay to wrapped bales. It was assumed that the first dry bales fed to both cattle groups were second-cut, continuing until all second-cut dry bales were consumed. Second-cut wrapped bales were evenly distributed among all wrapped bales fed throughout the duration of time cattle are fed stored feed, based on the fraction of each year's total number of wrapped bales that were recorded to be second-cut.

For the 2024 exposure scenario, the PFAS content of stored feed was also estimated directly from PFAS concentrations measured in cut hay samples collected during the 2023 growing season. Samples were collected from fields 2, 3, 6, 7, and 8, with 1-8 composite sample areas within each field depending on field size and the area that had been cut for hay (Figure S1). Sampled hay fields represented 78% of the total 2023 crop fed to the 2024 cattle. For fields where more than one composite sample was collected, average PFOS and PFDA concentrations were calculated weighted by the acreage of each sample area. Fields 4, 9, 10, and 11 were also sourced for feed for the 2024 cattle (22% of the total 2023 crop), though hay samples were not collected from these fields. For these fields, the previously described approach for estimating PFAS concentrations in first-cut hay was applied. Both estimated and average measured PFAS concentrations in hay in each field were then weighted by the fraction of total feed sourced from each field to yield an overall weighted average PFAS concentration in stored feed to apply in the 2024 exposure scenario.

Grazing Exposure

Grazing exposure was modeled using farmer-reported order of rotation through pastures, measured fieldwide composite soil concentrations for each pasture, and soil-to-plant transfer factors to estimate PFAS in pasture forage. Exact dates cattle were rotated onto each pasture were not known and were estimated either by information provided by the farmer or based on relative acreage. Site-specific plant transfer factors representative of first-cut hay were applied for pasture forage, except for Field 8, which was grazed after at least one cutting of hay for stored feed, and thus, grazed forage was presumed to be representative of a second cutting. Pasture rotation order, estimated rotation dates, and plant transfer factors applied for each grazing event are provided for the first year of life in Table S5 and for the second year of life in Table S6.

Table S5. Pasture rotation schedule in cattle first year of life and plant transfer factors applied to estimate PFAS in pasture forage in each field

Pasture	Start Date	End Date	PFOS Plant Transfer Factor	PFDA Plant Transfer Factor
13	Birth	Jul 29	0.094	0.026
14	Jul 30	Sep 14	0.094	0.026
8	Sep 15	Oct 31	0.254	0.055

Table S6. Pasture rotation schedule in cattle second year of life and plant transfer factors applied to estimate PFAS in pasture forage in each field

Pasture	Start Date	End Date	PFOS Plant Transfer Factor	PFDA Plant Transfer Factor
15	May 10	Jun 13	0.094	0.026
12	Jun 14	Oct 31	0.094	0.026

Incidental soil ingestion was considered while cattle were grazing at a soil intake rate of 4% dry matter intake based on US EPA's soil ingestion rate utilized in a risk assessment model for beef cattle raised on contaminated soil [8]. This rate aligns with the highest mean soil intake rate observed in a USDA study for cattle kept on land with sparse vegetation [9].

Farm Management Analyses

Economic Analysis

Table S7. Summary of economic analysis comparing net profit per animal of raising cattle to 24 months to slaughter for sale of meat, or raising cattle to 18 months for live auction sale.

Scenario	Live weight (lb)[a]	Meat weight (lb)[b]	Price per pound	Gross profit per animal	Total Cost of Clean Hay[e]	Total Cost of Grain[f]	Net Profit Per Animal
1. Slaughter	1113	578	$4.73[c]	$2,733.94	$351.12	$342.36	$2,040.46
2. Live auction	969	-	$1.49[d]	$1,443.81	-	-	$1,443.81

[a] Estimated using growth equations from the DE_PopTK model and described by Mikkonen et. al [10].
[b] Calculated assuming hanging weight is 63% of live weight, and 17.5% of hanging weight accounts for weight of bones [11,12].
[c] National average monthly retail price data per pound of 100% ground beef obtained from Federal Reserve Economic Data and averaged for years 2020 through 2024 [13].
[d] Monthly average auction prices for feeder cattle obtained from USDA National Agricultural Statistics Service (NASS) and averaged for years 2020 through 2024 [14].
[e] Annual average hay prices per ton in Maine obtained from USDA NASS Quick Stats averaged for years 2020 through 2024 [15]. Total cost of hay calculated assuming 2.1 tons of hay per animal fed over a 6 month period based on a daily forage intake rate of 2.5% body weight [16].
[f] Based on study farm records for purchased corn meal and grain for 5 animals in 2022. Total cost of purchased grain was divided by 5 to yield total cost of grain per animal.

Timed feeding simulations

The DE_PopTK model was applied to evaluate PFAS muscle levels if farm-sourced feed was optimized to allocate feed containing the lowest PFOS concentrations to beef cattle intended for sale or slaughter and fed in order of highest to lowest PFOS concentrations in feed. Two simulations were run to model implementing this management strategy during 1) the first winter of life for cattle to be sold at live auction at 18 months, and 2) the second winter of life for cattle raised for slaughter at 24 months. To model exposure in these simulations, first the number of bales sourced from each hay field was averaged over the four years for which farm records were provided, as shown in Table S8. For these simulations, all feed was assumed to be first-cut. The farmer previously reported feeding cattle raised for beef at a rate of 1-2 bales per week. As a conservative estimate, it was assumed that bales were fed to both beef animals and breeding stock at a rate of one bale every three days. Based on this rate, it was determined that beef cattle would require 63 bales during the typical 6-month period that cattle consume stored feed. Based on PFOS concentrations measured in hay field soils and the average number of bales sourced from each field, bales were selected from fields with lowest PFOS levels as needed to meet a total of 63 bales to be fed out in order of highest to lowest PFOS levels over the 6-month period. PFAS concentrations in feed were estimated using site-specific plant transfer factors as described above. The model was run to evaluate both PFOS and the sum of PFOS and PFDA levels in muscle tissue.

Table S8. Average number of hay bales sourced from each field or group of fields over the 2019-2023 period.

Hay Field(s)[a]	Average Number of Bales Sourced from Field(s)
1<br>2<br>3	43
4	25
6<br>7	89
8	83
9	10
10<br>11	12

[a] Number of hay bales were recorded in some years as a mix from one or more fields. These fields were grouped in order to calculate an average number of bales over the four-year period where records were available.

PFAS Concentrations in Soil and Forage

Table S9. PFOS and PFDA concentrations in fieldwide composite soil samples from farm hay fields and pastures

Hay Field	Acreage	Field Use	Soil Sampling Method	PFOS in Soil (ng/g dw)	PFDA in Soil (ng/g dw)
1	5.03	Hay	15 Grid	9.20	65.2
2	14.90	Hay	30 Grid[a]	8.35 ± 0.82	81.2 ± 7.60
3	1.01	Hay	15 Grid	12.0	111
4	6.59	Hay	15 Grid	8.17	92.0
5	1.68	Hay	15 Grid	0.351	0.618
6	7.68	Hay	15 Grid	11.6	93.1
7	12.46	Hay	30 Grid[a]	19.8 ± 1.59	90.3 ± 4.16
8	13.58	Hay/Pasture	30 Grid[a]	14.9 ± 0.49	89.8 ± 3.78
9	2.63	Hay	15 Grid	1.02	2.76
10	2.53	Hay	15 Grid	0.394	0.652
11	2.32	Hay	15 Grid	0.359	0.640
12	12.32	Pasture	10 Part Composite	ND[b]	ND[c]
13	10.08	Pasture	10 Part Composite	0.551	1.03
14	10.4	Pasture	10 Part Composite	0.311	0.353
15	2.99	Pasture	10 Part Composite	0.676	0.769

[a] Results provided for 30 grid cell composite soil samples are the mean and standard deviation of three replicates.

[b] PFOS method detection limit of 0.2 ng/g

[c] PFDA method detection limit of 0.1 ng/g

Table S10. PFOS and PFDA measured in composite cut hay samples

Field	Sample Date	Field Composite Sample Area	Sample Area Acreage	PFOS (ng/g wet weight)	PFOS (ng/g dry weight)[a]	PFDA (ng/g wet weight)	PFDA (ng/g dry weight)[a]
2	28-Sep-23	2A	1.44	3.21	3.77	14.5	17.0
		2B	1.16	4.00	4.71	12.7	14.9
		2C	0.79	1.55	1.83	20.4	24.0
3	6-Sep-23	3A	0.75	5.86	6.89	21.1	24.8
6	6-Sep-23	6A	0.90	1.81	2.13	16.0	18.8
		6B	0.87	5.80	6.82	16.5	19.4
		6C	0.79	5.42	6.38	14.6	17.2
7	25-Jul-23	7A	1.29	2.83	3.33	6.95	8.18
		7B	1.26	6.26	7.36	10.9	12.9
		7C	1.26	4.98	5.86	9.24	10.9
		7D	1.23	5.01	5.90	6.86	8.07
		7E	1.13	8.60	10.1	13.7	16.1
		7F	0.98	6.80	8.00	7.98	9.39
		7G	0.96	13.3	15.7	12.8	15.1
		7H	0.99	9.77	11.5	12.2	14.4
8	23-Aug-23	8A	0.88	8.29	9.76	17.7	20.8
		8B	1.25	7.27	8.55	13.0	15.2
		8C	0.42	0.30	0.35	0.80	0.94
		8D	1.13	7.37	8.67	14.2	16.7
		8E	1.98	6.97	8.21	14.7	17.3

[a] Moisture content was not measured in these samples. Concentrations on a dry matter basis were estimated using an assumed percent moisture of 15%, based on the mean percent moisture measured in previous hay samples collected from the study farm.

Study Farm Biomonitoring Results

Table S11. PFOS and PFDA measured in 2021 frozen beef sample

Animal ID	Slaughter Date	Muscle (ng/g)	
		PFOS	PFDA
1	21-Apr-21	2.87	4.29

Table S12. PFOS and PFDA measured in 2022 depuration study

Animal ID	Sample Date	Muscle (ng/g)		Serum (ng/mL)		Plasma (ng/mL)	
		PFOS	PFDA	PFOS	PFDA	PFOS	PFDA
1	7-Mar-22	2.71	4.35	28.2	43.9	20.0	33.2
	18-Mar-22	-	-	32.0	48.2	20.6	32.8
	14-Apr-22	-	-	20.2	23.6	19.8	22.3
	26-May-22	0.50	0.35 J	17.0	19.3	15.3	19.0
2	7-Mar-22	2.18	2.98	24.7	33.8	16.1	24.1
	18-Mar-22	-	-	22.9	26.6	21.3	24.7
	14-Apr-22	-	-	17.5	18.2	14.5	15.4
	26-May-22	0.43	ND	13.6	14.5	12.2	13.2
3	7-Mar-22	1.95	2.82	25.2	37.9	19.0	29.0
	18-Mar-22	-	-	28.5	34.4	24.4	29.7
	14-Apr-22	-	-	19.5	25.6	18.1	22.5
	26-May-22	0.70	0.97	16.1	19.9	15.4	19.6
4	7-Mar-22	2.09	3.29	24.0	33.7	20.1	31.7
	18-Mar-22	-	-	32.4	32.6	29.0	28.9
	14-Apr-22	-	-	21.4	24.9	16.9	19.2
	26-May-22	0.97	1.23	15.8	16.9	14.8	17.0
5	7-Mar-22	2.34	3.08	20.7	29.6	21.8	31.7
	18-Mar-22	-	-	10.8	16.3	16.6	25.9
	14-Apr-22	-	-	-	-	-	-
	26-May-22	0.69	0.64	14.9	17.1	14.5	16.3

Table S13. PFOS and PFDA measured in 2023 cattle serum

Animal ID	Sample Date	Serum (ng/mL)	
		PFOS	PFDA
1	28-Jun-23	9.84	10.1
	18-Sep-23	12.0	14.3
2	28-Jun-23	14.3	17.5
	18-Sep-23	16.3	18.5
3	28-Jun-23	-	-
	18-Sep-23	13.8	17.6
4	28-Jun-23	-	-
	18-Sep-23	10.6	11.9
5	28-Jun-23	12.5	16.1
	18-Sep-23	16.2	18.0
6	28-Jun-23	-	-
	18-Sep-23	11.3	16.5

Table S14. PFOS and PFDA measured in 2024 cattle serum

Animal ID	Sample Date	Serum (ng/mL)	
		PFOS	PFDA
1	30-Apr-24	92.7	198
	5-Sep-24	19.6	21.7
2	30-Apr-24	98.3	214
	5-Sep-24	26.5	29.3
3	30-Apr-24	84.1	190
	5-Sep-24	19.4	17.2
4	30-Apr-24	86.2	195
	5-Sep-24	20.3	21.4
5	30-Apr-24	91.9	189
	5-Sep-24	11.7	10.2
6	30-Apr-24	98.8	219
	5-Sep-24	21.5	25.5
7	30-Apr-24	74.9	179
	5-Sep-24	12.7	12.1
8	30-Apr-24	85.9	235
	5-Sep-24	18.3	24.7

Off-site Biomonitoring Results

Table S15. PFOS and PFDA measured in paired serum and plasma samples in beef cattle used to derive serum:plasma ratios

Farm	Animal #	Serum (ng/mL)		Plasma (ng/mL)		Serum:Plasma Ratio	
		PFOS	PFDA	PFOS	PFDA	PFOS	PFDA
A	1	34.4	22.8	32.6	21.8	1.055	1.046
	2	25.4	16.4	26.5	16.7	0.958	0.982
	3	28.0	20.6	25.0	17.4	1.120	1.184
	4	29.6	17.9	38.6	24.3	0.767	0.737
	5	29.1	16.3	30.3	19.4	0.960	0.840
B	1	28.2	43.9	20.0	33.2	1.410	1.322
	2	24.7	33.8	16.1	24.1	1.534	1.402
	3	25.2	37.9	19.0	29.0	1.326	1.307
	4	24.0	33.7	20.1	31.7	1.194	1.063
	5	20.7	29.6	21.8	31.7	0.950	0.934
C	1	150	3.30	144	3.14	1.042	1.051
	2	121	5.99	117	5.56	1.034	1.077
	3	171	8.00	176	7.98	0.972	1.003
	4	114	4.27	113	3.95	1.009	1.081
	5	146	4.48	136	4.30	1.074	1.042
	6	226	14.4	219	13.9	1.032	1.036
	7	163	5.63	165	5.73	0.988	0.983
	8	219	6.12	231	6.07	0.948	1.008
	9	66.1	1.82	70.2	1.95	0.942	0.933
	10	223	10.4	233	10.8	0.957	0.963
	11	95.1	3.89	96.2	4.04	0.989	0.963
	12	102	2.17	95.5	2.01	1.068	1.080

Table S16. Paired muscle and serum PFDA concentrations in beef and dairy cattle used for meta-mean analysis derivation of a PFDA muscle:serum partition coefficient.

Farm Name	Animal #	Cattle Type[a]	Muscle (ng/g)		Serum (ng/mL)		Muscle:Serum Partition Coefficient	
			PFOS	PFDA	PFOS	PFDA	PFOS	PFDA
A	1	Beef	2.51	1.90	34.4	22.8	0.07	0.083
	2	Beef	1.93	1.21	25.4	16.4	0.08	0.074
	3	Beef	3.13	2.23	28.0	20.6	0.11	0.108
	4	Beef	3.01	2.06	29.6	17.9	0.10	0.115
	5	Beef	1.82	1.10	29.1	16.3	0.06	0.067
B	1	Beef	1.74	3.45	28.2	43.9	0.06	0.079
	2	Beef	2.28	3.89	24.7	33.8	0.09	0.115
	3	Beef	1.66	3.05	25.2	37.9	0.07	0.080
	4	Beef	1.25	2.28	24.0	33.7	0.05	0.068
	5	Beef	2.06	3.64	20.7	29.6	0.10	0.123
C[b]	1	Beef	18.9	1.93	463	30.9	0.04	0.062
	2	Beef	24.0	2.06	372	34.2	0.06	0.060
	3	Beef	22.0	1.12	407	31.2	0.05	0.036
	4	Beef	13.2	1.13	281	40.9	0.05	0.028
	5	Dairy	133	3.52	2449	48.2	0.05	0.073
	6	Dairy	57.3	1.23	633	20.9	0.09	0.059
	7	Dairy	113	2.62	1019	34.7	0.11	0.076
	8	Dairy	96.3	2.15	842	32.9	0.11	0.065
	9	Dairy	83.1	2.82	741	38.5	0.11	0.073
	10	Dairy	56.0	1.17	1632	58.6	0.03	0.020
	11	Dairy	37.5	0.63	1113	29.9	0.03	0.021
	12	Dairy	35.6	0.71	615	19.4	0.06	0.037
	13	Dairy	67.2	1.71	853	35.1	0.08	0.049
	14	Dairy	26.0	0.53	492	17.4	0.05	0.030
	15	Dairy	82.8	2.11	908	45.5	0.09	0.046
	16	Dairy	46.5	1.16	312	14.7	0.15	0.079
	17	Dairy	36.1	5.11	509	120	0.07	0.042
	18	Dairy	36.7	0.80	742	29.7	0.05	0.027
	19	Dairy	20.7	ND	690	32.1	0.03	-
D	1	Beef	0.659	ND	15.0	ND	0.044	-
	2	Beef	5.60	ND	56.6	ND	0.099	-
	3	Beef	3.15	ND	35.6	ND	0.088	-

[a] Muscle:serum partitioning was similar between beef and dairy animals; therefore, these data were pooled when available.

[b] Only plasma data were available at this farm. Plasma concentrations were adjusted to estimated serum concentrations using a weighted average serum:plasma ratio of 1.060 for PFOS and 1.047 for PFDA, calculated from Table S15.

Muscle:Serum Partition Coefficients

Study or Subgroup	Mean	SD	Total	Mean IV, Random, 95% CI
subgroup = PFDA				
Green Bounty 2022	0.09	0.0210	5	0.09 [0.07; 0.11]
Ledgerock 2022	0.09	0.0240	5	0.09 [0.07; 0.11]
Tozier 2021	0.05	0.0200	18	0.05 [0.04; 0.06]
Total (95% CI)			28	0.08 [0.01; 0.14]
Heterogeneity: $Tau^2 = 0.0006$; $Chi^2 = 24.84$, df = 2 ($P < 0.01$); $I^2 = 92\%$				
subgroup = PFOS				
Green Bounty 2022	0.08	0.0210	5	0.08 [0.07; 0.10]
Ledgerock 2022	0.07	0.0210	5	0.07 [0.06; 0.09]
Tozier 2021	0.07	0.0330	19	0.07 [0.06; 0.08]
Drew 2021 (d63)	0.07	0.0180	12	0.07 [0.06; 0.08]
Drew 2021 (d215)	0.08	0.0280	5	0.08 [0.06; 0.10]
Drew and Hagen 2020	0.13	0.0400	6	0.13 [0.10; 0.16]
Kowalczyk 2013	0.09	0.0138	6	0.09 [0.08; 0.10]
Vestergren 2013	0.19	.	5	0.19
Arndt 2022	0.08	0.0290	3	0.08 [0.04; 0.11]
Total (95% CI)			66	0.08 [0.07; 0.09]
Heterogeneity: $Tau^2 = < 0.0001$; $Chi^2 = 15.51$, df = 7 ($P = 0.03$); $I^2 = 55\%$				

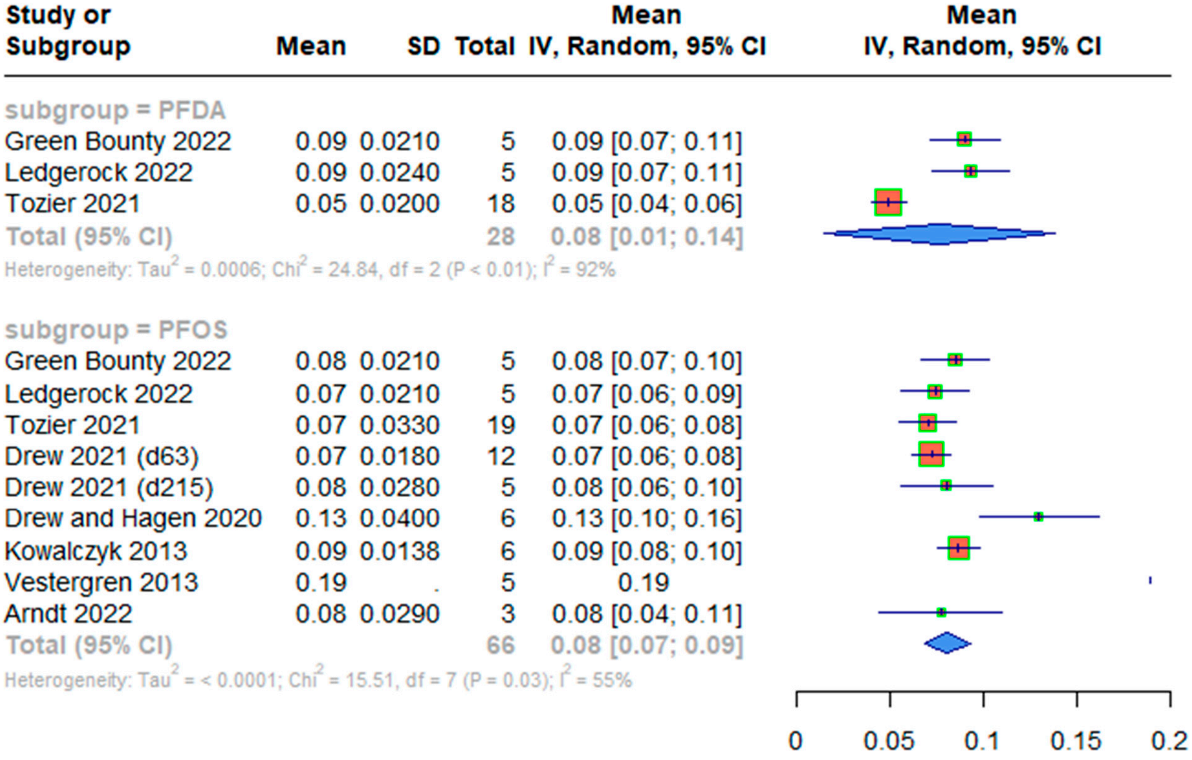

Figure S2. Forest plots depicting mean PFOS and PFDA muscle:serum partition coefficients. CI is confidence interval and IV is instrumental variable. Meta-analyses methods utilized are described in Mikkonen, et al. [10] supplementary materials.

Exposure Model Inputs

Table S17. 2021 Scenario exposure model inputs

Exposure Pathway	Cattle Age (mo.)	Parameter	PFOS	PFDA
Grazing (ng/g)	0-1.5	Field 13 Soil	0.55	1.03
		Field 13 Pasture	0.05	0.03
	1.5-3	Field 14 Soil	0.31	0.35
		Field 14 Pasture	0.03	0.01
	3-4.5	Field 8 Soil	14.9	89.8
		Field 8 Pasture[a]	3.8	4.9
Stored Feed (ng/g)	4.5-11	Estimated 2019 1st Cut Hay	1.2	2.1
Grazing (ng/g)	11-12	Field 15 Soil	0.68	0.77
		Field 15 Pasture	0.06	0.02
	12-16.5	Field 12 Soil[b]	0.17	0.17
		Field 12 Pasture	0.02	0.004
Stored Feed (ng/g)[c]	16.5-22.5	Estimated 2020 1st Cut Hay	1.2	2.2
		Estimated 2020 2nd Cut Hay	1.2	1.6
Water (ng/L)	0-22.5	Untreated well water	1.3	0

[a] Pasture forage in field 8 was assumed to be representative of a second cutting and PFAS concentrations were calculated using estimated 2nd cut plant transfer factors.

[b] PFOS and PFDA were not detected in soil in field 12; a value of ½ the LOR was imputed to estimate levels in soil and resulting levels in forage.

[c] Feeding rates of 1st and 2nd cut hay were estimated using methods described on pg. 7. PFAS concentrations in 2nd cut hay were calculated using estimated 2nd cut plant transfer factors.

Table S18. 2022 Scenario exposure model inputs

Exposure Pathway	Cattle Age (mo.)	Parameter	PFOS	PFDA
Grazing (ng/g)	0-1.5	Field 13 Soil	0.55	1.03
		Field 13 Pasture	0.05	0.03
	1.5-4	Field 14 Soil	0.31	0.35
		Field 14 Pasture	0.03	0.01
	4-5.5	Field 8 Soil	14.9	89.8
		Field 8 Pasture[a]	3.8	4.9
Stored Feed (ng/g) [b]	5.5-12	Estimated 2020 1st Cut Hay	1.2	2.2
		Estimated 2020 2nd Cut Hay	1.2	1.6
Grazing (ng/g)	12-13	Field 15 Soil	0.68	0.77
		Field 15 Pasture	0.06	0.02
	13-17.5	Field 12 Soil[c]	0.17	0.17
		Field 12 Pasture	0.02	0.004
Stored Feed (ng/g) [b]	17.5-20	Estimated 2021 1st Cut Hay	1.3	2.3
		Estimated 2021 2nd Cut Hay	2.8	3.2
	20-24.5	Clean Feed	0	0
Water (ng/L)	0-21	Untreated well water	1.3	0
	21-24.5	Treated well water	0	0

[a] Pasture forage in field 8 was assumed to be representative of a second cutting and PFAS concentrations were calculated using estimated 2nd cut plant transfer factors.

[b] Feeding rates of 1st and 2nd cut hay were estimated using methods described on pg. 7. PFAS concentrations in 2nd cut hay were calculated using estimated 2nd cut plant transfer factors.

[c] PFOS and PFDA were not detected in soil in field 12; a value of ½ the LOR was imputed to estimate levels in soil and resulting levels in forage.

Table S19. 2023 Scenario exposure model inputs

Exposure Pathway	Cattle Age (mo.)	Parameter	PFOS	PFDA
Grazing (ng/g)	0-2	Field 13 Soil	0.55	1.03
		Field 13 Pasture	0.05	0.03
	2-3.5	Field 14 Soil	0.31	0.35
		Field 14 Pasture	0.03	0.01
	3.5-5	Field 8 Soil	14.9	89.8
		Field 8 Pasture[a]	3.8	4.9
Stored Feed (ng/g)	5-11.5	Estimated 2022 1st Cut Hay	1.3	2.2
Grazing (ng/g)	11.5-12.5	Field 15 Soil	0.68	0.77
		Field 15 Pasture	0.06	0.02
	12.5-17	Field 12 Soil[b]	0.17	0.17
		Field 12 Pasture	0.02	0.004
Water (ng/L)	0-17	Treated well water	0	0

[a] Pasture forage in field 8 was assumed to be representative of a second cutting and PFAS concentrations were calculated using estimated 2nd cut plant transfer factors.

[b] PFOS and PFDA were not detected in soil in field 12; a value of ½ the LOR was imputed to estimate levels in soil and resulting levels in forage.

Table S20. 2024 Scenario exposure model inputs

Exposure Pathway	Cattle Age (mo.)	Parameter	PFOS	PFDA
Grazing (ng/g)	0-2	Field 13 Soil	0.55	1.03
		Field 13 Pasture	0.05	0.03
	2-3.5	Field 14 Soil	0.31	0.35
		Field 14 Pasture	0.03	0.01
	3.5-5	Field 8 Soil	14.9	89.8
		Field 8 Pasture[a]	3.8	4.9
Stored Feed (ng/g)[b]	5-11.5	Estimated 2023 1st Cut Hay	1.3	2.1
		Measured 2023 Hay	5.9	11.7
Grazing (ng/g)	11.5-12.5	Field 15 Soil	0.68	0.77
		Field 15 Pasture	0.06	0.02
	12.5-17	Field 12 Soil[c]	0.17	0.17
		Field 12 Pasture	0.02	0.004
Water (ng/L)	0-17	Treated well water	0	0

[a] Pasture forage in field 8 was assumed to be representative of a second cutting and PFAS concentrations were calculated using estimated 2nd cut plant transfer factors.

[b] Two model runs were performed to compare model results using 1) estimated PFAS concentrations in 2023 hay based on site-specific plant transfer factors and 2) PFAS measured in composite samples collected from the 2023 hay crop.

[c] PFOS and PFDA were not detected in soil in field 12; a value of ½ the LOR was imputed to estimate levels in soil and resulting levels in forage.

Additional Model Runs

Sensitivity Analysis

Insufficient experiemental data are available at present to directly estimate a volume of distribution (Vd) for PFDA in beef cattle. A PFOS Vd value previously derived by Mikkonen, et al. [10] was assumed for PFDA. To address uncertainty in this parameter value, a sensitivity analysis was performed to evaluate model sensitivity to a 2-fold increase and 2-fold decrease in mean Vd while maintaining a constant coefficient of variation of 16.5%. This analysis was performed using the 2024 scenario data, as this was the dataset where PFAS was measured in feed, eliminating uncertainty surrounding estimating soil-to-plant transfer. Stochastic simulations were separately run with the differing distributions for a PFDA Vd with all other parameter inputs remaining unchanged. Results of this analysis (Figure S3) indicate that a Vd centered at 0.043 L/kg results in the model overpredicting serum results as compared to measured serum levels. Conversely, increasing the Vd to 0.17 L/kg results in mixed results with the model having a tendency to underpredict at the observation representing the end of the accumlation period, whereas better agreement was achieved at the observation respresenting depuration. Collectively, the sensitivity analysis suggests that a Vd for PFDA is unlikely to be more than 2-fold different than the assumed Vd of 0.085 L/kg.

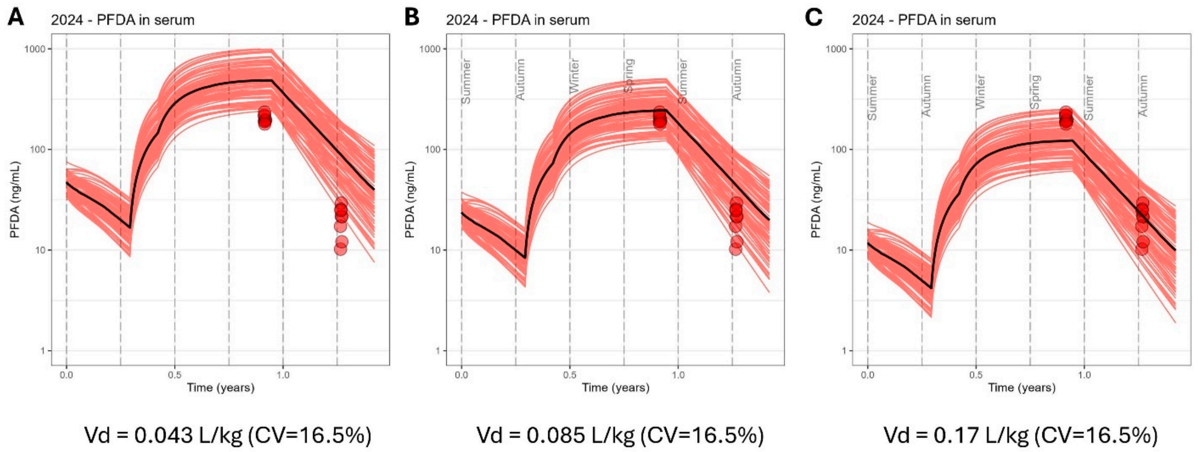

Figure S3. Volume of distribution sensitivity analysis results where A) mean PFDA Vd is decreased by 2-fold, B) mean PFDA Vd is equal to mean PFOS Vd, and C) mean PFDA Vd is increased by 2-fold. Results demonstrate model sensitivity to changes in Vd, and indicate that estimated PFDA Vd is likely to within 2-fold of PFOS Vd.

Timed Feeding Simulations

Based on PFOS concentrations measured in hay field soils and the average number of bales recorded to be harvested from each of the farm's hay fields, all of the bales from fields 4 and 9-11, and 16 bales from fields 1-3 were selected to source feed for beef cattle raised for sale or slaughter in timed feeding simulations. Bales from each field or group of fields were fed out according to the schedule provided in Table S21. Simulation results for timed feeding in the first year of life (Figure S4) compared to timed feeding in the second year of life (Figure S5) are provided for PFOS and the sum of PFOS and PFDA.

Table S21. Schedule of timed feeding of bales selected for beef cattle raised for sale or slaughter, and estimated PFAS concentrations in selected feed.

Dates	Field(s)	Number of bales fed	PFOS in Soil (ng/g)	Estimated PFOS in Feed (ng/g dw)	PFDA in Soil (ng/g)	Estimated PFDA in Feed (ng/g dw)
Nov 1 - Dec 19	1, 2, 3[a]	16	8.73	0.82	78.77	2.05
Dec 20 - Mar 3	4	25	8.17	0.77	92.00	2.39
Mar 4 - Apr 2	9	10	1.02	0.10	2.76	0.07
Apr 3 - May 9	10, 11[a]	12	0.38	0.04	0.65	0.02

[a] Number of hay bales were recorded in some years as a mix from multiple fields. These fields were grouped in order to calculate an average number of bales over the four year period where records were available. For these grouped fields, weighted average soil PFAS concentrations were calculated using PFAS soil levels measured in each field weighted by the acreage of each field.

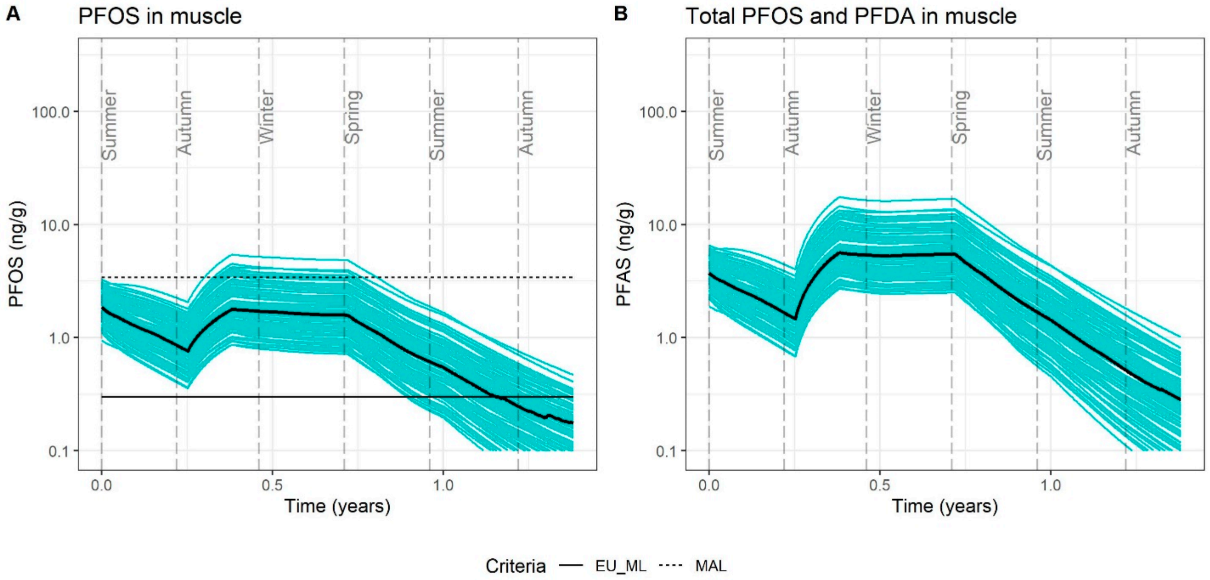

Figure S4. Management practice simulations in which feed sourced from farm hay fields with lowest PFOS soil concentrations (ranging from 0.4 to 8.4 ng/g) was allocated to cattle intended to be sold at live auction at around 18 months of age, with timed feeding from highest to lowest soil levels during their first winter of life and prior to their second season of grazing. Estimated concentrations in muscle over the lifespan of cattle are presented for A) PFOS and B) the sum of PFOS and PFDA.

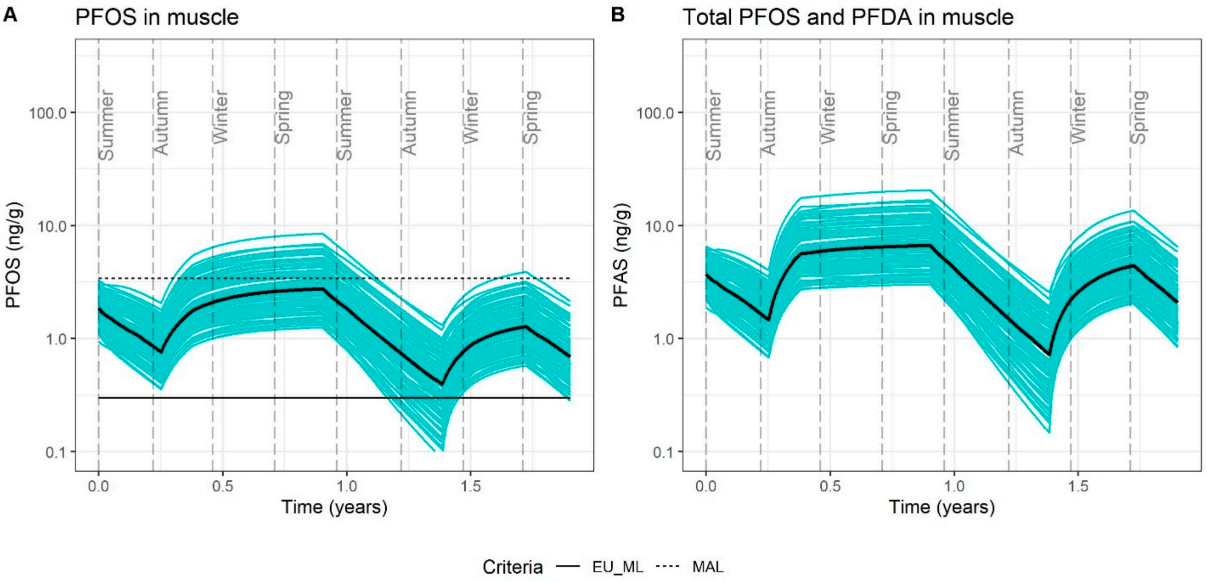

Figure S5. Management practice simulations in which feed sourced from farm hay fields with lowest PFOS soil concentrations (ranging from 0.4 to 8.4 ng/g) was allocated to cattle intended to be slaughtered at around 24 months of age, with timed feeding from highest to lowest soil levels during their second winter of life prior to slaughter in spring. Estimated concentrations in muscle over the lifespan of cattle are presented for A) PFOS and B) the sum of PFOS and PFDA.

Alternative model run for 2024 exposure scenario using estimated PFAS in feed

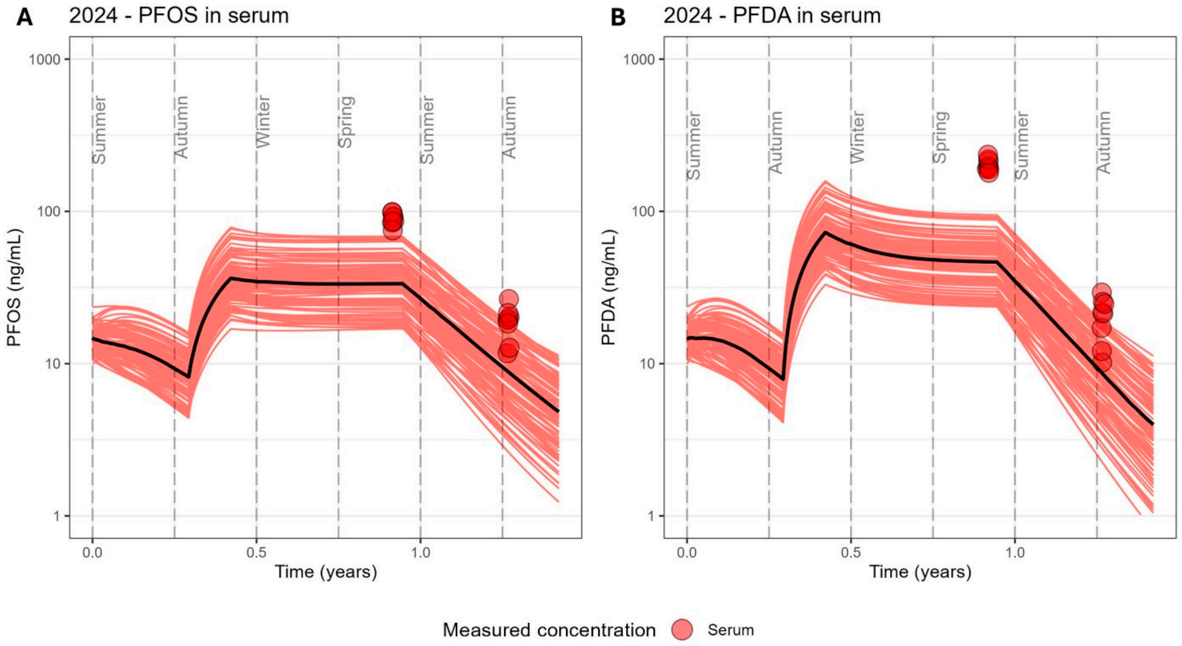

Figure S6. Alternative model run for the 2024 exposure scenario based on estimated PFAS levels in feed calculated using site-specific plant transfer factors.

References

1. Simones, T.L.; Evans, C.; Goossen, C.P.; Kersbergen, R.; Mallory, E.B.; Genualdi, S.; Young, W.; Smith, A.E. Uptake of Per- and Polyfluoroalkyl Substances in Mixed Forages on Biosolid-Amended Farm Fields. *Journal of Agricultural and Food Chemistry* **2024**, *72*, 23108-23117, doi:10.1021/acs.jafc.4c02078.
2. Genualdi, S.; Young, W.; DeJager, L.; Begley, T. Method Development and Validation of Per- and Polyfluoroalkyl Substances in Foods from FDA's Total Diet Study Program. *J Agric Food Chem* **2021**, *69*, 5599-5606, doi:10.1021/acs.jafc.1c01777.
3. U.S. FDA. Foods Program Compendium of Analytical Laboratory Methods. *Determination of 16 Perfluoroalkyl and Polyfluoroalkyl Substances (PFAS) in Food using Liquid Chromatography-Tandem Mass Spectrometry (LC-MS/MS)* **2021**, *2024*.
4. U.S. EPA. Method 1633, Revision A Analysis of Per- and Polyfluoroalkyl Substances (PFAS) in Aqueous, Solid, Biosolids, and Tissue Samples by LC-MS/MS **2024**.
5. USDA Food Safety and Inspection Service. CLG-PFAS 2.04 Effective: 02/28/23 Screening, Determination, and Confirmation of PFAS by UHPLC-MS-MS. **2023**.
6. Weyrauch, K.; Ochoa, C.; Matsuda, R.; Duverna, R.; Lenov, I. A Survey of the Levels of 16 Per- and Polyfluoroalkyl Substances in Meat, Chicken, and Siluriformes Fish, 2019 to 2023. *Food Protection Trends* **2025**, *45*, 154, doi:10.4315/FPT-24-045.
7. MDIFW and Maine CDC. PFAS in Deer and Turkeys in the Fairfield Area, Maine. **2024**.
8. U.S. EPA. Preliminary Remediation Goals for Radionuclides (PRG). Available online: https://epa-prgs.ornl.gov/radionuclides/ (accessed on October 29, 2024).
9. Fries, G.F.; Marrow, G.S.; Snow, P.A. Soil Ingestion by Dairy Cattle. *Journal of Dairy Science* **1982**, *65*, 611-618, doi:https://doi.org/10.3168/jds.S0022-0302(82)82238-8.
10. Mikkonen, A.T.; Martin, J.; Upton, R.N.; Moenning, J.-L.; Numata, J.; Taylor, M.P.; Roberts, M.S.; Mackenzie, L. Dynamic exposure and body burden models for per- and polyfluoroalkyl substances (PFAS) enable management of food safety risks in cattle. *Environment International* **2023b**, *180*, 108218, doi:https://doi.org/10.1016/j.envint.2023.108218.
11. USDA. 5 Area Weekly Weighted Average Direct Slaughter Cattle (January 2023-December 2024). Available online: https://mymarketnews.ams.usda.gov/viewReport/2477 (accessed on January 13, 2025).
12. University of Maine Cooperative Extension. Bulletin #1071, What To Expect When Buying A Freezer Beef. Available online: https://extension.umaine.edu/publications/1071e/ (accessed on January 13, 2025).
13. Federal Reserve Economic Data. Table Data - Average Price: Ground Beef, 100% Beef (Cost per Pound/453.6 Grams) in U.S. City Average. Available online: https://fred.stlouisfed.org/data/APU0000703112 (accessed on Febuary 7, 2025).
14. USDA National Agricultural Statistics Service. Prices Received: Cattle Prices Received by Month, US. Available online: www.nass.usda.gov/Charts_and_Maps/Agricultural_Prices/priceca.php (accessed on January 13, 2025).
15. USDA National Agricultural Statistics Service. 2024 State Agriculture Overview Maine. Available online: www.nass.usda.gov/Quick_Stats/Ag_Overview/stateOverview.php?state=MAINE (accessed on January 13, 2025).
16. Oklahoma State University Extension. Nutrient Requirements of Beef Cattle E-974. **2023**.
